# Optimal Dietary Patterns for Lower Weight Gain and Risk of Obesity Surrounding Menopause

**DOI:** 10.1001/jamanetworkopen.2026.13102

**Published:** 2026-05-20

**Authors:** Tong Xia, Danielle E. Haslam, A. Heather Eliassen, JoAnn E. Manson, Qi Sun, Walter C. Willett, Shilpa N. Bhupathiraju, Cuilin Zhang, Frank B. Hu

**Affiliations:** 1 Channing Division of Network Medicine, Department of Medicine, Brigham and Women’s Hospital, Harvard Medical School, Boston, Massachusetts; 2 Department of Nutrition, Harvard T.H. Chan School of Public Health, Boston, Massachusetts; 3 Department of Epidemiology, Harvard T.H. Chan School of Public Health, Boston, Massachusetts; 4 Division of Preventive Medicine, Department of Medicine, Brigham and Women’s Hospital, Harvard Medical School, Boston, Massachusetts; 5 Global Centre for Asian Women’s Health, Yong Loo Lin School of Medicine, National University of Singapore, Singapore; 6 Department of Obstetrics and Gynaecology, Yong Loo Lin School of Medicine, National University of Singapore, Singapore; 7 Bia-Echo Asia Centre for Reproductive Longevity and Equality, Yong Loo Lin School of Medicine, National University of Singapore, Singapore

## Abstract

**Importance:**

Weight gain is common during menopause, and healthy dietary patterns are key to its management. However, effectiveness of different diets for weight management during this period remains unclear.

**Objective:**

To examine and compare associations of multiple dietary patterns with weight gain and obesity risk in the years surrounding menopause.

**Design, Setting, and Participants:**

This prospective, population-based cohort study included women observed over a 12-year period surrounding menopause in the Nurses’ Health Study II (1989-2019). Data analysis was performed between November 2024 and May 2025.

**Exposures:**

Diet was assessed every 4 years using validated food frequency questionnaires. Dietary scores included the plant-based diet index (PDI), healthy PDI, unhealthy PDI, Mediterranean diet, Dietary Approaches to Stop Hypertension, Planetary Health Diet Index (PHDI), low-carbohydrate diet (LCD), healthy LCD, unhealthy LCD, empirical dietary inflammatory pattern, empirical dietary index for hyperinsulinemia (EDIH), and ultraprocessed food intake.

**Main Outcomes and Measures:**

The outcomes were annual changes in self-reported body weight (kilograms per year) and incident obesity. Generalized estimating equations were used to estimate annual weight change across dietary patterns. Cox proportional hazards models were used to estimate risk of obesity across dietary patterns.

**Results:**

Among 38 283 women (mean [SD] age, 45.6 [3.0] years), the mean (SD) weight gain was 0.80 (1.00) kg per year. During 340 122 person-years of follow-up, 5214 women developed obesity. After adjusting for age, race and ethnicity, marital status, income, postmenopausal hormone therapy use, parity, smoking, alcohol, energy intake, physical activity, and baseline body mass index, the reverse EDIH (quintile 5 vs 1) was associated with the largest reduction in weight gain (mean, −0.28 kg/y; 95% CI, −0.30 to −0.26 kg/y). For incident obesity, the lowest risk was observed for the PHDI (hazard ratio, 0.46; 95% CI, 0.42 to 0.51) and reverse EDIH (hazard ratio, 0.51; 95% CI, 0.46 to 0.56). EDIH showed the largest positive correlations with red or processed meats, sodium, and French fries. PHDI showed the largest positive correlations with nuts, unsaturated fats, whole grain carbohydrates, and vegetable protein.

**Conclusions and Relevance:**

In this prospective cohort study of women during menopause, adopting low-insulinemic and planetary health diets, low in red and processed meats, sodium, potatoes, and French fries and rich in nuts, legumes, fruits, vegetables, and whole grains, was associated with optimized weight management.

## Key Points

**Question:**

Are dietary patterns associated with long-term weight change and obesity risk among women during the years surrounding menopause, and which patterns best support weight management?

**Findings:**

In a cohort study of 38 283 Nurses’ Health Study II participants, low-insulinemic and planetary-health dietary patterns, lower in red or processed meats, sodium, potatoes, and French fries, and higher in plant foods, were associated with the lowest weight gain and risk of obesity.

**Meaning:**

These findings suggest that low-insulinemic, planetary-health diets may optimize weight management during menopause and, if promoted during routine midlife care, could improve women’s weight management and long-term cardiometabolic health.

## Teaser

This cohort study examines the associations of multiple dietary patterns with weight gain and obesity risk during menopause.

## Introduction

The menopausal transition is characterized by hormonal and metabolic changes that increase adiposity, alter fat distribution, and elevate obesity risk.^1,2,3,4^ These changes elevate risks of type 2 diabetes, cardiovascular disease (CVD), and certain cancers.^5,6,7^ Diet quality and composition represent key modifiable determinants of weight gain and cardiometabolic health. Randomized clinical trials^8,9,10^ show that plant-based diets, Dietary Approaches to Stop Hypertension (DASH), and reduced ultraprocessed food (UPF) intake promote short-term weight loss. Cohort studies^11,12,13,14,15,16^ support long-term associations of dietary patterns with weight change and obesity.

Despite these advances, evidence on dietary patterns and weight management during menopause remains limited. Although studies have investigated individual dietary components in relation to weight changes during menopause,^1,17^ research on overall dietary patterns is sparse. Clarifying these associations may help inform dietary strategies to reduce weight gain, obesity, and cardiometabolic risk in women during menopause.

Moreover, few studies, either in general populations or during menopause, have directly compared multiple dietary patterns within the same cohort, leaving no empirical evidence for which pattern performs best at weight management. We addressed this menopause-specific gap by examining and comparing associations of 11 dietary patterns, including the plant-based dietary index (PDI), healthy plant-based diet index (hPDI), unhealthy plant-based diet index (uPDI), Mediterranean diet (MedDiet), DASH, Planetary Health Diet Index (PHDI), low-carbohydrate diet (LCD), healthy low-carbohydrate diet (HLCD), unhealthy low-carbohydrate diet (ULCD), empirical dietary inflammation pattern (EDIP), and empirical dietary index for hyperinsulinemia (EDIH), and UPF intake, with weight gain and obesity risk surrounding menopause in the Nurses’ Health Study II (NHS II) cohort.

## Methods

### Study Population

The NHS II enrolled 116 429 female registered nurses aged 25 to 42 years in 1989.^18^ We used data from 1989 to 2019, focusing on approximately 12 years around menopause, defined as 6 years before and after the cycle when menopause was first reported (eFigure 1 in Supplement 1). Menopause was determined when participants reported cessation of menstruation for at least 1 year. Weight change was assessed across six 2-year intervals spanning 3 questionnaires before and after menopause. The window of 6 years before and after menopause was selected on the basis of Stages of Reproductive Aging Workshop plus 10 staging and hormone patterns and ensured at least 2 dietary assessments before and after menopause^19^ (eMethods in Supplement 1). For weight change analyses, we excluded women with nonnatural or missing menopause type, age at menopause younger than 45 years, or those missing data on menopause age, interval data, missing height, prevalent cancer, CVD, or diabetes, or dietary patterns. Additional exclusions for weight change and obesity analyses ae shown in eMethods, eTable 1, eFigure 2, and eFigure 3 in Supplement 1. The study was approved by the institutional review boards of Brigham and Women’s Hospital and Harvard T.H. Chan School of Public Health; questionnaire return constituted informed consent, and reporting followed Strengthening the Reporting of Observational Studies in Epidemiology (STROBE) reporting guidelines for cohort studies.

### Diet Assessment

Diet was assessed using validated semiquantitative food frequency questionnaires in 1991 and every 4 years thereafter, where participants reported average intakes of specified portion sizes for more than 130 food items.^20,21^ Several dietary patterns were evaluated to capture different dimensions of diet quality. Although PDI, hPDI, DASH, MedDiet, and PHDI generally reflect higher intakes of fruits, vegetables, whole grains, nuts, and legumes and lower intakes of red and processed meats, each emphasizes distinct components, and correlation analyses suggested they are not interchangeable (eFigure 4 in Supplement 1). PDI assigns positive scores to plant foods and reverse scores to animal foods; hPDI emphasizes healthy plant foods, whereas uPDI emphasizes less-healthy plant foods.^22^ DASH represents a dietary pattern designed for blood pressure control, emphasizing lower sodium intake.^23^ MedDiet reflects a Mediterranean-style dietary pattern rich in olive oil and fish.^23^ PHDI reflects a plant-forward EAT–Lancet reference diet emphasizing sustainability and cardiometabolic health.^24^ LCD scores rank macronutrient distribution; HLCD emphasizes vegetable protein and unsaturated fats with fewer refined carbohydrates, whereas ULCD emphasizes animal protein and saturated fats with fewer high-quality carbohydrates.^14^ EDIH^25^ and EDIP^26^ are empirically derived dietary scores reflecting the insulinemic and inflammatory potential of diet, respectively. UPF intake was assessed using the NOVA classification.^27^ Full definitions and scoring details are provided in eTables 2 and 3 in Supplement 1.

### Weight Change Assessment

Height was self-reported at baseline. Weight was self-reported biennially with high validity compared with technician measurements.^28^ Annualized weight change (kilograms per year) was calculated for each 2-year interval as the difference in weight between consecutive questionnaires divided by the interval length. Thus, each participant contributed repeated interval-specific weight-change measures across follow-up. Body mass index (BMI) was calculated as weight in kilograms divided by height in meters squared. Obesity was defined as BMI greater than or equal to 30.

### Covariates Assessment

Covariates were assessed biennially using validated questionnaires, including age, race and ethnicity (categorized in 4 groups: Asian, American Indian or Alaska Native, and Native Hawaiian or Other Pacific Islander; Black; Hispanic; and White), marital status, smoking status, postmenopausal hormone use (PMH), parity, and physical activity (PA). Race and ethnicity were included as a covariate to account for potential confounding. Annual household income, collected in 2011, was used as a proxy for long-term income. Alcohol (grams per day) and total caloric intake (kilocalories per day) were derived from food frequency questionnaires every 4 years.

### Statistical Analysis

Data analysis was performed between November 2024 and May 2025. For weight change analysis, dietary scores were averaged across 12 years spanning menopause to assess habitual diet, decrease within-subject variation, and reflect long-term diet, a standard approach in nutritional epidemiology.^29^ Diet scores were assessed in quintiles and per SD. Adjusted mean annualized weight change (kilograms per year) was estimated using generalized estimating equations with repeated measures to account for within-person correlation across follow-up intervals, specifying an unstructured working correlation matrix. Each participant contributed up to six 2-year interval observations. Separate models were fit for each dietary pattern score. Models were adjusted for age (≤45 vs >45 years), race and ethnicity, marital status (never vs ever), annual household income (<$50 000, $50 000-$99 000, ≥$100 000, or missing), PMH (never, ever, or missing), parity (nulliparous, 1 and 2, or ≥3 births), smoking (never vs ever), alcohol intake (grams per day), total energy intake (less than vs greater than or equal to the median), PA (<1000 vs ≥1000 metabolic equivalent of task, minutes per week), and interval-specific baseline BMI (>18 to <25 vs ≥25). Alcohol was not adjusted for MedDiet, EDIP, and EDIH. To facilitate comparisons across dietary patterns, uPDI, LCD, ULCD, EDIP, EDIH, and UPF were reverse-coded so that higher values consistently indicated healthier patterns. Primary association estimates were obtained from separate one-diet pattern models. Differences between dietary patterns were calculated as contrasts of quintile 5 vs 1 estimates. Pairwise *z *tests were conducted to evaluate *P* values for differences between quintile 5 vs 1 regression coefficients, using coefficients and the variance–covariance matrix from models jointly including the 2 dietary scores. We examined food groups and dietary patterns correlations using Spearman coefficients and associations of food groups with weight gain as in the main analysis. In secondary analyses, we stratified by premenopausal or postmenopausal status, repeated models in 4-year and 8-year windows around menopause, and assessed interval-specific weight change across six 2-year intervals, testing diet by interval interactions with Wald χ^2^ tests. We also conducted an exploratory analysis of weight loss proportions across dietary patterns.

Cox proportional hazards models were used to estimate hazard ratios (HRs) and 95% CIs for incident obesity across dietary quintiles and per SD during the 12-year window. Participants were followed up from baseline to incident obesity, death, cancer, CVD, type 2 diabetes, loss to follow-up, or end of follow-up. Dietary intakes were averaged from baseline to development of obesity or censoring. Models adjusted for age, race and ethnicity, marital status, income, PMH, parity, smoking, alcohol, total energy intake, PA, and baseline BMI, excluding alcohol for MedDiet, EDIP, and EDIH. For cross-pattern comparisons, unhealthy scores were reverse-coded so higher values indicated healthier patterns. Primary associations (quintile 5 vs 1) were estimated in separate one-pattern models, and differences in quintile 5 vs 1 log(HR) estimates were tested using pairwise *z* tests based on 2-pattern models and their variance–covariance matrices. We also examined food groups and dietary patterns correlations and their associations with obesity risk.

Stratified analyses for diet and weight change were conducted by age, BMI, PA, smoking, alcohol, and PMH. Effect modification was tested using Wald χ^2^ test. We conducted sensitivity analyses by averaging dietary scores within each interval to assess associations with weight change, and adjusting for PA tertiles as covariates for weight change and obesity. Analyses used SAS statistical software version 9.4 (SAS Institute) and R statistical software version 4.2.1 (R Project for Statistical Computing) with 2-sided *P* values. Multiple testing was controlled by Bonferroni correction within each outcome family (12 tests each for weight change and incident obesity) and across weight change interaction tests (12 × 6 = 72 tests); Bonferroni-adjusted *P* < .05 was considered significant.

## Results

### Population Characteristics

Among 38 283 women (mean [SD] age, 45.6 [3.0] years), the mean (SD) weight gain was 0.80 (1.00) kg per year. During 340 122 person-years of follow-up, 5214 women developed obesity. Those with higher DASH, MedDiet, PHDI, HLCD, or low ULCD, EDIH, and UPF scores were generally older, exercised more, and had lower baseline weight and BMI (eTable 4 in Supplement 1). eFigure 5 in Supplement 1 presents weight gain over 12 years surrounding menopause, with more pronounced increase before menopause.

### Dietary Patterns and Weight Change

**Figure 1.**
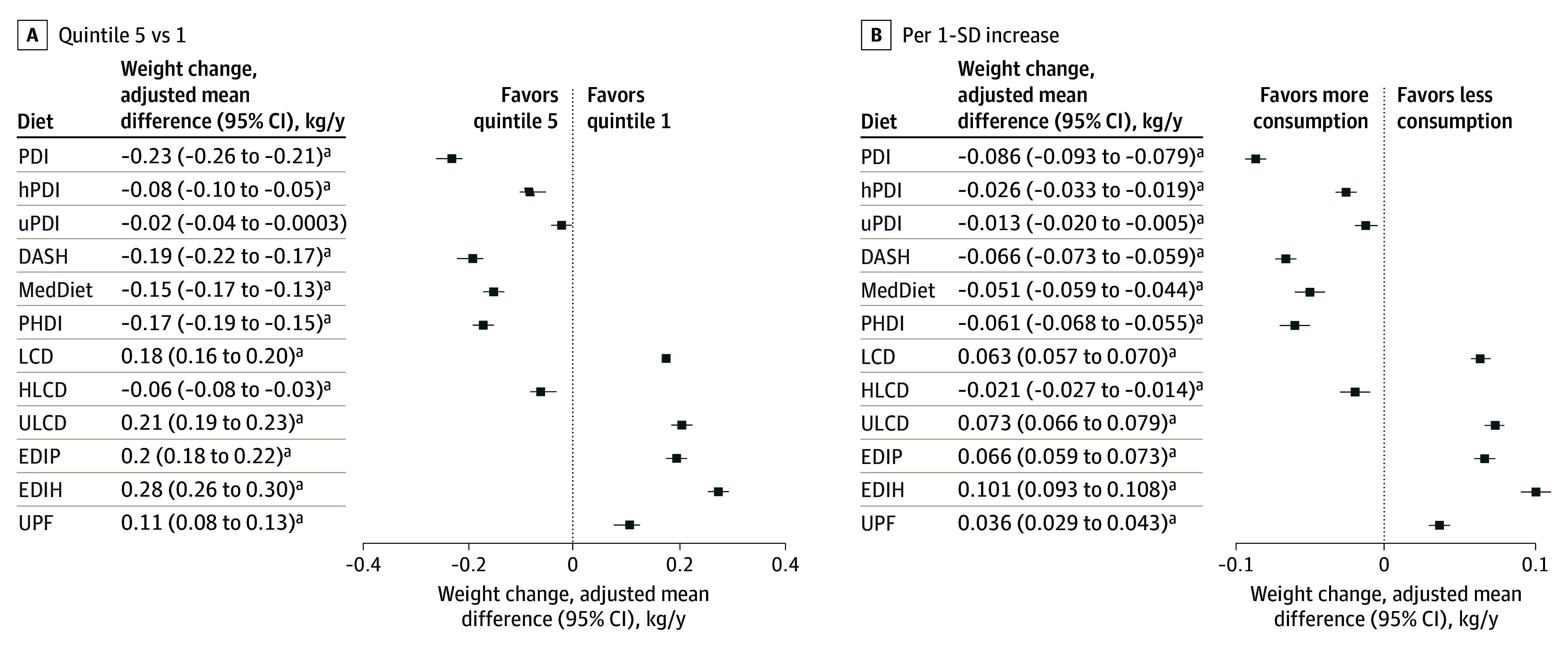
Dot Plots of Associations Between Dietary Patterns and Weight Gain Graphs show adjusted mean annualized weight change (kilograms per year) for quintile 5 vs quintile 1 (A) and per 1-SD increase (B). Changes were estimated using generalized estimating equations with repeated measures to account for within-person correlation across follow-up intervals, specifying an unstructured working correlation matrix. Models were adjusted as described in the Statistical Analysis section. DASH indicates Dietary Approaches to Stop Hypertension; HLCD, healthy low-carbohydrate diet; hPDI, healthy plant-based diet index; LCD, low-carbohydrate diet; MedDiet, Mediterranean diet; PDI, plant-based diet index; PHDI, Planetary Health Diet Index; ULCD, unhealthy low-carbohydrate diet; uPDI, unhealthy plant-based diet index; and UPF, ultraprocessed food. ^a^Bonferroni-adjusted *P* < .05.

After multivariable adjustment, higher score on healthy diets (PDI, hPDI, DASH, MedDiet, PHDI, and HLCD) were associated with reduced weight gain, while higher scores on unhealthy diets (LCD, ULCD, EDIP, EDIH, and UPF) were associated with more weight gain (Figure 1; eTable 5 in Supplement 1). Similar trends were seen for continuous scores (per SD) (Figure 1; eTable 6 in Supplement 1). Although categorical uPDI (quintile 5 vs 1) was not significant, continuous uPDI showed a modest inverse association with weight gain. When comparing across pattern scores, among significant associations, the reduction in annual weight gain with diet quintile (quintile 5 vs 1) ranged from −0.28 kg/y for reverse EDIH to −0.06 kg/y for HLCD. Higher reverse EDIH showed the largest reduction for weight gain (mean, −0.28 kg/y; 95% CI, −0.30 to −0.26 kg/y) (eFigure 6 in Supplement 1). Findings were consistent across premenopausal and postmenopausal strata and within 4-year and 8-year windows around menopause (eFigure 7 in Supplement 1). eFigure 8 in Supplement 1 shows interval-specific estimates across 12-year windows, with broadly parallel quintile 5 vs 1 differences over time; no diet and interval interaction met the significance threshold, supporting the use of the 12-year averaged estimates. For healthier patterns (eg, hPDI, aMED, DASH, PHDI, and HLCD), a higher proportion of women had weight loss in quintile 5 vs 1; for unhealthy patterns (eg, LCD, ULCD, EDIP, and EDIH), the opposite was observed (eTable 7 in Supplement 1).

**Figure 2.**
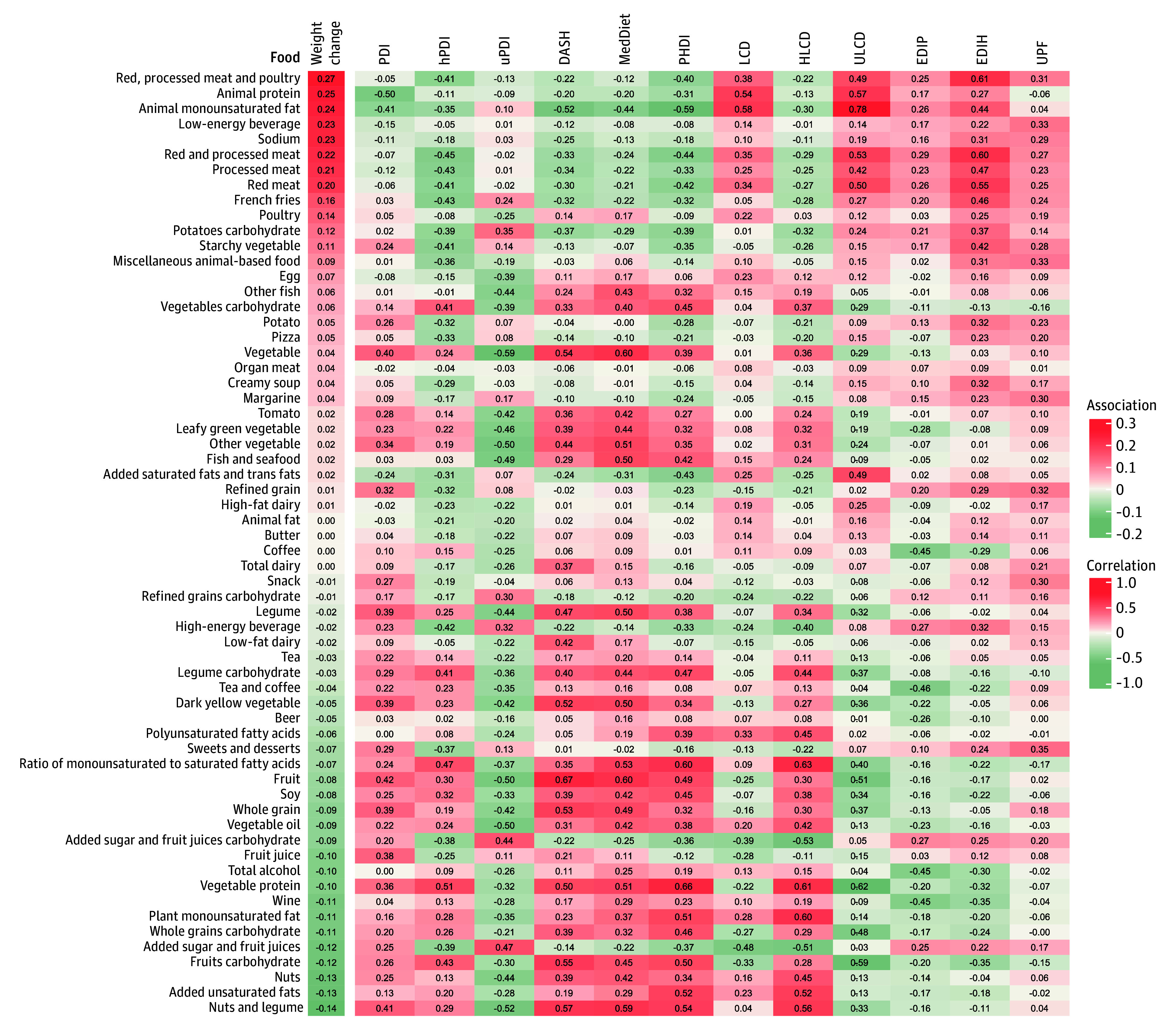
Heat Map of Spearman Correlations Between Dietary Patterns and Food Groups, and Associations Between Food Groups and Weight Gain The associations of food groups (quintile 5 vs 1) with weight gain are indicated on the left of the figure. The Spearman correlations between dietary patterns and food groups are indicated on the right of the figure. Adjusted mean annualized weight change (kilograms per year) was estimated using generalized estimating equations with repeated measures to account for within-person correlation across follow-up intervals, specifying an unstructured working correlation matrix. Models were adjusted as described in the Statistical Analysis section. DASH indicates Dietary Approaches to Stop Hypertension; EDIH, empirical dietary index for hyperinsulinemia; EDIP, empirical dietary inflammation pattern; HLCD, healthy low-carbohydrate diet; hPDI, healthy plant-based diet index; LCD, low-carbohydrate diet; MedDiet, Mediterranean diet; PDI, plant-based diet index; PHDI, Planetary Health Diet Index; ULCD, unhealthy low-carbohydrate diet; uPDI, unhealthy plant-based diet index; and UPF, ultra-processed food.

Figure 2 shows food group correlations with dietary patterns and associations with weight change over 12 years surrounding menopause. Red and processed meats and poultry, animal monounsaturated fatty acids (MUFA) and protein, low-energy drink, sodium, French fries, and potato were positively associated with weight gain, whereas nut, legume, unsaturated fats, fruits carbohydrate, plant MUFA, wine, and whole grains carbohydrate were protective against weight gain. The EDIH displayed positive correlations with several obesogenic foods, such as red or processed meats and poultry, low-energy beverages, sodium, French fries, potatoes, pizza, and cream soup. It also had inverse correlations with fruit carbohydrate, wine, vegetable protein, whole grain carbohydrate, and plant MUFA.

### Dietary Patterns and Obesity Risk

**Figure 3.**
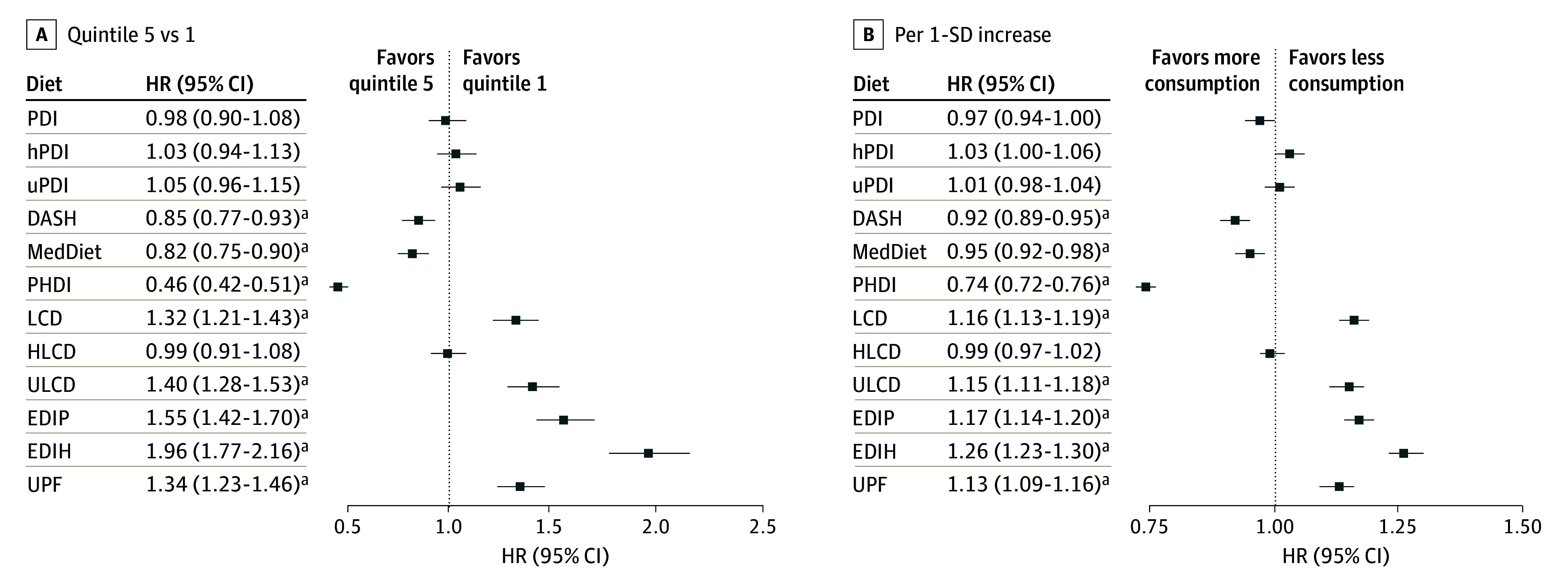
Dot Plot of Associations Between Dietary Patterns and Obesity Graphs show associations for quintile 5 vs quintile 1 (A) and per 1-SD increase (B). Cox proportional hazards model was used to calculate hazard ratios (HRs) and 95% CIs. Models were adjusted as described in the Statistical Analysis section. DASH indicates Dietary Approaches to Stop Hypertension; EDIH, empirical dietary index for hyperinsulinemia; EDIP, empirical dietary inflammation pattern; HLCD, healthy low-carbohydrate diet; hPDI, healthy plant-based diet index; LCD, low-carbohydrate diet; MedDiet, Mediterranean diet; PDI, plant-based diet index; PHDI, Planetary Health Diet Index; ULCD, unhealthy low-carbohydrate diet; uPDI, unhealthy plant-based diet index; and UPF, ultra-processed food. ^a^Bonferroni-adjusted *P* < .05.

After adjusting for age, race and ethnicity, marital status, income, PMH, parity, smoking status, alcohol, total energy intake, PA, and baseline BMI, healthy diets (DASH, MedDiet, and PHDI) were associated with lower risk of obesity, while unhealthy diets (LCD, ULCD, EDIP, EDIH, and UPF) were associated with an increased risk (Figure 3; eTable 8 in Supplement 1). Findings from continuous score analyses were similar (Figure 3; eTable 9 in Supplement 1). When reversing unhealthy diets, as shown in eFigure 9 in Supplement 1, HRs for obesity with diet quintile (quintile 5 vs 1) ranged from 0.46 (95% CI, 0.42-0.51) for PHDI to 0.85 (95% CI, 0.77-0.93) for DASH, among significant associations. Higher PHDI (HR, 0.46; 95% CI, 0.42-0.51) and reverse EDIH (HR, 0.51; 95% CI, 0.46-0.56) were associated with the lowest obesity risk.

**Figure 4.**
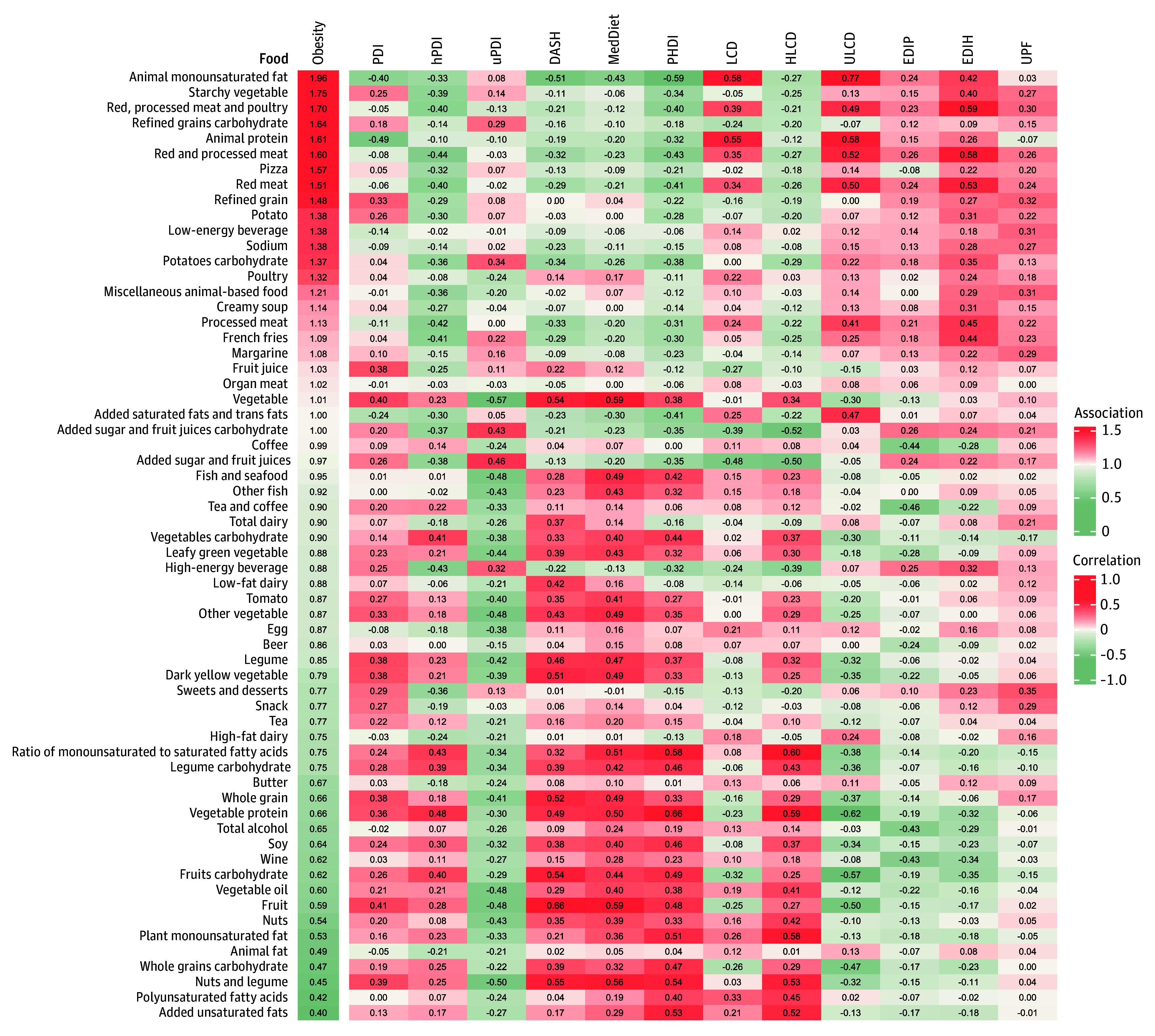
Heat Map of Spearman Correlations Between Dietary Patterns and Food Groups, and Associations Between Food Groups and Obesity Risk The associations of food groups (quintile 5 vs 1) with obesity risk are indicated on the left of the figure. The Spearman correlations between dietary patterns and food groups are indicated on the right of the figure. Models were adjusted as described in the Statistical Analysis section. DASH indicates Dietary Approaches to Stop Hypertension; EDIH, empirical dietary index for hyperinsulinemia; EDIP, empirical dietary inflammation pattern; HLCD, healthy low-carbohydrate diet; hPDI, healthy plant-based diet index; LCD, low-carbohydrate diet; MedDiet, Mediterranean diet; PDI, plant-based diet index; PHDI, Planetary Health Diet Index; ULCD, unhealthy low-carbohydrate diet; uPDI, unhealthy plant-based diet index; and UPF, ultra-processed food.

Figure 4 displays food group correlations with diet patterns and associations with obesity, consistent with Figure 2. The PHDI showed positive correlations with vegetable protein, nuts and legumes, unsaturated oils, fruit, and whole grain carbohydrate. It also showed inverse correlations with animal MUFA, red and processed meats and poultry, and refined grains carbohydrate.

### Sensitivity and Subgroup Analyses

In sensitivity analyses, results were unchanged when averaging dietary scores within each interval (eTable 10 in Supplement 1) or adjusting for PA tertiles (eTable 11 in Supplement 1). eFigure 10 and eTable 12 in Supplement 1 present stratified analyses of diet and weight change. The association between EDIP and weight change was significantly larger among PMH users.

## Discussion

In this large prospective cohort of perimenopausal women, healthy dietary patterns were associated with less weight gain and lower obesity risk, while unhealthy patterns showed the reverse pattern. To our knowledge, this is the first study to comprehensively compare multiple dietary patterns in relation to weight change and obesity risk during menopause within the same cohort. Across the dietary indices examined, several healthy patterns (eg, hPDI, MedDiet, DASH, and PHDI) shared common core features that appear promising for weight management during menopause, including higher intakes of fruits, vegetables, whole grains, nuts, and legumes, and lower intakes of red and processed meats, potatoes, French fries, and sodium. In contrast, unhealthy dietary patterns (eg, uPDI, EDIP, and EDIH) showed the opposite pattern of food-group correlations. Despite overlap in food components, differences in scoring algorithms and food-group weighting help explain heterogeneity in the strength of associations with weight change and obesity across dietary patterns. Notably, EDIH demonstrated the greatest association with weight gain, and the greatest associations with obesity were observed for PHDI and EDIH.

EDIH and EDIP, empirically derived dietary pattern scores reflecting insulinemic and inflammatory potential of diet, were associated with greater weight gain and obesity risk during menopause, extending NHS findings^15^ to perimenopausal women. There is ongoing debate about whether hyperinsulinemia precedes obesity.^30^ Our analyses showed consistent harmfulness of EDIH in both normal-weight and overweight women, supporting that elevated insulin secretion may play a role in weight gain.^30^ Among all dietary patterns examined, EDIH showed the greatest positive correlations with weight-promoting foods such as red and processed meats, sodium, French fries, potatoes, and cream soup. These foods may promote adiposity through multiple biological mechanisms, including impaired insulin sensitivity, enhanced adipocyte lipid storage signaling, hepatic lipogenesis, and altered fat partitioning.^15^ EDIH showed greater associations with increased weight gain than EDIP, consistent with prior findings in the NHS cohort.^15^ Although both EDIH and EDIP showed similar directions in food group correlations, EDIH had larger positive correlations with obesogenic foods like red and processed meat and French fries, whereas EDIP showed slightly larger inverse correlations with protective foods such as nuts and legumes. Notably, associations for weight gain with weight-promoting foods were larger than those with protective ones. In subgroup analyses, the association between EDIP and weight gain appeared larger among PMH users. This is biologically plausible given that hormone therapy can influence inflammatory pathways and adiposity regulation, potentially amplifying the metabolic effects of a proinflammatory dietary pattern.^31^

Similarly, although PHDI overlaps with other plant-forward dietary patterns, its stronger emphasis on high-quality plant protein and lower correlation with animal-derived and refined carbohydrate sources may explain its comparatively larger inverse association with obesity risk. Compared with other diets, PHDI had the highest positive correlation with protective components, including nuts and legumes, unsaturated oils, whole grain carbohydrates, plant MUFA, soy, and vegetable protein. It also had the lowest correlation with animal MUFA, red and processed meats and poultry, and potato carbohydrate. Those may explain why the greatest inverse associations for obesity were observed for high PHDI. No prior US study has examined PHDI’s link to weight or obesity, although findings from other populations are mixed (inverse in UK^32^ and Brazil^33^ but null in Finland,^34^ likely due to low adherence). In this first US cohort, higher PHDI was related to lower weight gain and the lowest obesity risk.

PDI and hPDI were associated with less weight gain, consistent with NHS findings.^11^ These observational findings were supported by randomized trial evidence in postmenopausal women showing that a healthy, low-fat, vegan diet can induce short-term weight loss compared with no dietary change.^35^ The inverse association between uPDI and weight gain was modest and should be interpreted cautiously. The uPDI scoring scheme assigns lower scores to several protein-rich foods, including nuts, legumes, soy, and animal products, because only less healthy plant foods are scored positively, whereas healthy plant foods and all animal foods are reverse scored (eTables 2 and 3 in Supplement 1).^22^ Consistent with this structure, uPDI was inversely correlated with animal, vegetable, and total protein intake in our data (eTable 13 in Supplement 1), and participants in the highest vs lowest uPDI quintile also had lower intakes of these proteins (eTable 14 in Supplement 1). Given that loss of lean mass is common during the menopausal transition^1,2,3,4^ and may be exacerbated by inadequate protein intake,^36^ differences in protein intake may partly contribute to the observed association. Notably, after additional adjustment for total protein intake, uPDI was positively associated with weight gain (eTable 15 in Supplement 1). However, body composition was not assessed in our study, and we could not distinguish fat mass from lean mass; therefore, this explanation should be interpreted cautiously. We extend evidence supporting DASH and Mediterranean diets for weight control^23,37,38,39^ to women during menopause. Consistent with NHS findings,^14^ ULCD was associated with greater weight gain, whereas HLCD was associated with less weight gain, highlighting that macronutrient source and carbohydrate quality matter. Vegetable protein and whole grains were associated with less weight gain, while red meat and poultry and refined carbohydrates were associated with more weight gain (Figures 2 and 4), suggesting that within low-carbohydrate (including animal-heavy ketogenic) patterns, diet quality and replacement choices may matter more than carbohydrate restriction alone. UPF intake was also associated with more weight gain and obesity, consistent with National Health and Nutrition Examination Survey evidence.^40^

### Strengths and Limitations

Tor our knowledge, this is the first long-term prospective study examining and comparing multiple dietary patterns with weight change and obesity across menopause. Strengths include the large sample, long-term follow-up, comprehensive adjustment for covariates, and robust findings across multiple sensitivity analyses.

Limitations include that food frequency questionnaires are subject to measurement error, even though they have been validated.^20,21^ In addition, self-reported weight is subject to error, even though it is highly correlated with measured weight.^28^ The study lacked detailed menopausal symptom data, temporally aligned sex-hormone measures, and sufficient clinical outcome such as incident type 2 diabetes, CVD, or cancer events during menopause for robust analyses. Body composition data were unavailable to distinguish fat from lean mass. Generalizability may be limited by the predominantly White health professional cohort, although this may enhance internal validity.

## Conclusions

The findings support low-insulinemic and planetary health diets, low in red and processed meats, potatoes, and sodium, and rich in nuts, legumes, fruits, vegetables, and whole grains, as an optimal strategy for weight management during menopause. Incorporating this dietary guidance into routine midlife care may help prevent obesity and support long-term cardiometabolic health in women.

## Notes

Supplement 1. eMethods. eTable 1. Numbers of exclusions & skips in each interval in weight change analysis eTable 2. Diet score definitions eTable 3. Calculation of the diet scores eTable 4. Characteristics of study population in the lowest and highest quintiles of dietary patterns eTable 5. Associations [β (95% CI)] of dietary patterns (Quintile 5 vs. 1) with weight change during menopause in the Nurse Health Study II eTable 6. Associations [β (95% CI)] of dietary patterns (per SD) with weight change during menopause in the Nurse Health Study II eTable 7. Proportion of women had weight loss in diet quintile 5 vs 1 eTable 8. Associations [HR (95% CI)] of dietary patterns (Quintile 5 vs. 1) with obesity risk during menopause in the Nurse Health Study II eTable 9. Associations [HR (95% CI)] of dietary patterns (per SD) with obesity risk during menopause in the Nurse Health Study II eTable 10. Associations of dietary patterns (quintile 5 vs. 1) with weight change during menopause in the Nurse Health Study II, diet assessed by average of two dietary scores within each weight change interval eTable 11. Associations of dietary patterns (quintile 5 vs. 1) with weight gain and obesity risk during menopause in the Nurse Health Study II, physical activity tertiles adjusted for eTable 12. Subgroup analyses of associations of dietary patterns (quintile 5 vs. 1) with weight gain during menopause in the Nurse Health Study II eTable 13. Correlations of dietary pattern scores with protein intake eTable 14. Mean protein intake (grams/day) in quintile 1 versus quintile 5 of each dietary pattern eTable 15. Association of dietary patterns (quintile 5 vs. 1) with weight gain after adjustment for total protein intake eFigure 1. Study timeline for weight change analysis around menopause eFigure 2. Sample selection of weight change analysis eFigure 3. Sample selection of obesity analysis eFigure 4. Pairwise Spearman correlations between dietary pattern scores eFigure 5. Weight (kg) and weight gain (kg/year) in each interval across 12 years around menopause eFigure 6. Associations between dietary patterns and weight gain, and comparison of weight change difference between all pairs of scores eFigure 7. Associations between dietary patterns and weight gain in women pre- and post-menopause, and within 4-year and 8-year around menopause eFigure 8. Annualized weight change by diet patterns in 12 years around menopause eFigure 9. Associations between dietary patterns and obesity risk, and comparison of difference of log hazard ratio of obesity between all pairs of scores eFigure 10. Associations between dietary patterns (quintile 5 vs. 1) and weight gain in subgroups eReferences

Supplement 2. Data Sharing Statement
