# Supplementary material for: Optimal Dietary Patterns for Lower Weight Gain and Risk of Obesity Surrounding Menopause
Source: JAMA Netw Open. 2026 May 20;9(5):e2613102. doi: 10.1001/jamanetworkopen.2026.13102 (PMC13191392; doi:10.1001/jamanetworkopen.2026.13102)
Supplement: Supplement 1. — eMethods. eTable 1. Numbers of exclusions & skips in each interval in weight change analysis eTable 2. Diet score definitions eTable 3. Calculation of the diet scores eTable 4. Characteristics of study population in the lowest and highest quintiles of dietary patterns eTable 5. Associations [β (95% CI)] of dietary patterns (Quintile 5 vs. 1) with weight change during menopause in the Nurse Health Study II eTable 6. Associations [β (95% CI)] of dietary patterns (per SD) with weight change during menopause in the Nurse Health Study II eTable 7. Proportion of women had weight loss in diet quintile 5 vs 1 eTable 8. Associations [HR (95% CI)] of dietary patterns (Quintile 5 vs. 1) with obesity risk during menopause in the Nurse Health Study II eTable 9. Associations [HR (95% CI)] of dietary patterns (per SD) with obesity risk during menopause in the Nurse Health Study II eTable 10. Associations of dietary patterns (quintile 5 vs. 1) with weight change during menopause in the Nurse Health Study II, diet assessed by average of two dietary scores within each weight change interval eTable 11. Associations of dietary patterns (quintile 5 vs. 1) with weight gain and obesity risk during menopause in the Nurse Health Study II, physical activity tertiles adjusted for eTable 12. Subgroup analyses of associations of dietary patterns (quintile 5 vs. 1) with weight gain during menopause in the Nurse Health Study II eTable 13. Correlations of dietary pattern scores with protein intake eTable 14. Mean protein intake (grams/day) in quintile 1 versus quintile 5 of each dietary pattern eTable 15. Association of dietary patterns (quintile 5 vs. 1) with weight gain after adjustment for total protein intake eFigure 1. Study timeline for weight change analysis around menopause eFigure 2. Sample selection of weight change analysis eFigure 3. Sample selection of obesity analysis eFigure 4. Pairwise Spearman correlations between dietary pattern scores eFigure 5. Weight (kg) and weight gain (kg/y [file jamanetwopen-e2613102-s001.pdf]

Supplemental Online Content

Xia T, Haslam DE, Eliassen AH, et al. Optimal dietary patterns and lower weight gain and obesity during menopause. *JAMA Netw Open*. 2026;9(5):e2613102. doi:10.1001/jamanetworkopen.2026.13102

eMethods
eTable 1. Numbers of exclusions & skips in each interval in weight change analysis
eTable 2. Diet score definitions
eTable 3. Calculation of the diet scores
eTable 4. Characteristics of study population in the lowest and highest quintiles of dietary patterns
eTable 5. Associations [β (95% CI)] of dietary patterns (Quintile 5 vs. 1) with weight change during menopause in the Nurse Health Study II
eTable 6. Associations [β (95% CI)] of dietary patterns (per SD) with weight change during menopause in the Nurse Health Study II
eTable 7. Proportion of women had weight loss in diet quintile 5 vs 1
eTable 8. Associations [HR (95% CI)] of dietary patterns (Quintile 5 vs. 1) with obesity risk during menopause in the Nurse Health Study II
eTable 9. Associations [HR (95% CI)] of dietary patterns (per SD) with obesity risk during menopause in the Nurse Health Study II
eTable 10. Associations of dietary patterns (quintile 5 vs. 1) with weight change during menopause in the Nurse Health Study II, diet assessed by average of two dietary scores within each weight change interval
eTable 11. Associations of dietary patterns (quintile 5 vs. 1) with weight gain and obesity risk during menopause in the Nurse Health Study II, physical activity tertiles adjusted for
eTable 12. Subgroup analyses of associations of dietary patterns (quintile 5 vs. 1) with weight gain during menopause in the Nurse Health Study II
eTable 13. Correlations of dietary pattern scores with protein intake
eTable 14. Mean protein intake (grams/day) in quintile 1 versus quintile 5 of each dietary pattern **eTable 15.** Association of dietary patterns (quintile 5 vs. 1) with weight gain after adjustment for total protein intake
eFigure 1. Study timeline for weight change analysis around menopause.
eFigure 2. Sample selection of weight change analysis.
eFigure 3. Sample selection of obesity analysis.
eFigure 4. Pairwise Spearman correlations between dietary pattern scores.
eFigure 5. Weight (kg) and weight gain (kg/year) in each interval across 12 years around menopause.
eFigure 6. Associations between dietary patterns and weight gain, and comparison of weight change difference between all pairs of scores.
eFigure 7. Associations between dietary patterns and weight gain in women pre- and post-menopause, and within 4-year and 8-year around menopause.
eFigure 8. Annualized weight change by diet patterns in 12 years around menopause.
eFigure 9. Associations between dietary patterns and obesity risk, and comparison of difference of log hazard ratio of obesity between all pairs of scores.
eFigure 10. Associations between dietary patterns (quintile 5 vs. 1) and weight gain in subgroups.
eReferences

This supplemental material has been provided by the authors to give readers additional information about their work.

© 2026 Xia T et al. JAMA Network Open.

eMethods

Rationale for 12 years around menopause (six years before and after the cycle in which menopause was first reported)

The six years prior final menstrual period (FMP) was chosen to approximate combined Stages of Reproductive Aging Workshop (STRAW)+10 transition stages −2 (variable duration) and stage −1 (approximately 1–3 years before the FMP)[1]. This mapping is also biologically supported by Study of Women's Health Across the Nation endocrine trajectories showing that follicle-stimulating hormone (FSH) begins rising as early as ~6 years before the FMP[2]. Thus, the window encompasses the period when endocrine dysregulation emerges and accelerates. The six years after the FMP encompass STRAW+10 stage +1a–+1c (early postmenopause, 5-6 years), during which FSH levels stabilize by roughly six years post-FMP[2] and early postmenopausal physiology consolidates[1]. Additionally, because diet was assessed every four years, this 12-year window ensured at least two diet measurements both before and after menopause. As no universally established pre/post window exists, we adopted this biologically grounded span and aligned it with prior Nurses' Health Study II work[3]. To evaluate robustness, we repeated analyses using narrower 4-year (±2 years) and 8-year (±4 years) windows and stratified by pre- and postmenopausal status (see Statistical Analysis section).

Full exclusions criteria

We excluded women with non-natural or missing menopause type (n=46,333), missing age at menopause (n=512), age at menopause <45 years (n=3,400), or missing data across intervals (n=4,727). Baseline exclusions also included women missing height (n=81), with prevalent cancer, cardiovascular disease (CVD), or diabetes (n=3,214), or missing dietary patterns (n=6,694), resulting in 51,468 eligible participants (**eFigure 2** in **Supplement 1**).

Weight change analyses included participants with non-missing weight data across intervals, excluding intervals with incident cancer, CVD, or diabetes. We also excluded those with weight loss >2.27 kg/year[3–5], weight gain >2.5 × interquartile range, or body mass index (BMI) ≤18 or ≥40 kg/m² to minimize bias from non-physiologic weight trajectories or underlying illness, consistent with prior studies on diet and weight change[3] (**eFigure 2** and **eTable 1** in **Supplement 1**). There were 38,283, 36,422, 36,420, 35,303, 33,154, and 31,111 participants in each of 6, 2-y weight-change intervals. For obesity incidence analyses, additional exclusions included those missing baseline BMI (n=5,854), baseline obesity (BMI ≥30 kg/m², n=10,829), underweight (BMI ≤18 kg/m², n=372), no follow-up (n=62), or missing dietary pattern data (n=5,063), yielding 35,982 participants (**eFigure 3** in **Supplement 1**).

© 2026 Xia T et al. JAMA Network Open.

eTable 1. Numbers of exclusions & skips in each interval in weight change analysis

**Interval-dependent exclusion:**	**x1-x2[1]**	**x2-x3**	**x3-x4**	**x4-x5**	**x5-x6**	**x6-x7**
Incident cancer or diabetes or cardiovascular diseases (exclude once diagnosis)	596	651	1,160	1,144	1,229	1,188
Missing weight change	6,645	7,840	7,122	6,232	7,768	9,085
Weight loss > 2.27 kg/y (i.e., 5 pounds/y)	3,857	3,913	3,708	4,475	3,997	3,674
Weight gains more than 2.5 × interquartile range	838	815	596	714	624	617
Underweight or morbidly obese (body mass index > 40 or < 18)	1,249	1,231	1,215	1,193	1,145	1,013
**N for each period**	38,283	36,422	36,420	35,303	33,154	31,111

[1] x1-x2 indicates the yearly weight change from questionnaire-x1 to questionnaire-x2.

© 2026 Xia T et al. JAMA Network Open.

eTable 2. Diet score definitions

Diet score	Range	Definition
PDI	18-90	The 18 food groups were categorized as healthy plant foods, less-healthy plant foods, or animal foods and were divided into quintiles of consumption; each quintile was assigned a score from 1 to 5. For the overall PDI, all plant food groups were scored positively (Q1=1 to Q5=5), whereas all animal food groups were reverse scored (Q1=5 to Q5=1)[6].
hPDI	18-90	For the hPDI, healthy plant food groups (whole grains, fruits, vegetables, nuts, legumes, vegetable oils, and tea/coffee) were scored positively, while less-healthy plant food groups (fruit juices, sugar-sweetened beverages, refined grains, potatoes, and sweets/desserts) and all animal food groups (animal fats, dairy, eggs, fish/seafood, meat [poultry and red meat], and miscellaneous animal-based foods) were reverse scored[6].
uPDI	18-90	For the uPDI, less-healthy plant food groups were scored positively, whereas healthy plant food groups and all animal food groups were reverse scored. Scores across the 18 food groups were summed to derive each index, with a theoretical range of 18 to 90. Additional details have been described previously[6].
DASH	8-40	1 to 5 points were assigned based on quintiles of intake in servings per day of fruits, vegetables, nuts and legumes, low-fat dairy products, and whole grains. Scoring was inverse for sodium, sugar-sweetened beverages and red and processed meat, with more points for lower consumption. More details were provided in a previous study[7].
MedDiet	0-9	The MedDiet score reflects food and nutrients common in traditional Mediterranean diets and awards 1 point for an intake greater than the cohort specific median for vegetables, legumes, fruits, nuts, whole grains, fish, and monounsaturated:saturated fat ratio and 1 point if intake was less than the cohort median for red and processed meat, and for alcohol intake between 5 and 15 g/d for women and 10–25 g/d for men[7].
PHDI	0-140	The PHDI quantifies adherence to the Ref diet included in the EAT-Lancet report by assessing the intake of 15 food groups, including whole grain, tubers, vegetable, whole fruit, dairy foods, red/processed meat, chicken and other poultry, eggs, fish and shellfish, nuts, nonsoy legumes, soybean/soy foods, added unsaturated oils (not including transfat), added saturated oils and transfat, added sugar and sugar from fruit juice[8].
LCD	0-30	Consumption of carbohydrate, fat, and protein was categorized into 11 levels based on each macronutrient's percentage contribution to total energy intake. Fat and protein were ranked in ascending order (0 for the lowest category to 10 for the highest), whereas carbohydrate was ranked in descending order (10 for the lowest category to 0 for the highest). These three ranks were summed to create the LCD[9].
HLCD	0-30	The HLCD was calculated by summing the ranks of vegetable protein and vegetable fat and the reversed rank of less healthful carbohydrates (from potatoes, added sugar, and refined grains)[9].
ULCD	0-30	The ULCD was calculated by summing the ranks of animal protein and animal fat and the reversed rank of more healthful carbohydrates (from non-starchy vegetables [excluding potatoes and French fries], fruits [excluding added sugar from fruit juice], legumes, and whole grains)[9].
EDIH	/	The EDIH score is a weighted sum of 18 food groups, with higher scores indicating higher insulinemic potential (predictive of fasting plasma C-peptide) of the whole diet and lower scores suggesting lower insulinemic potential[10].
EDIP	/	The EDIH score is a weighted sum of 18 food groups, with higher scores indicating higher inflammatory potential (predictive of interleukin-6, C-reactive protein (CRP), and tumor necrosis factor (TNF)α receptor 2) of the whole diet and lower scores suggesting lower inflammatory potential, identified from reduced-rank regression[11].
UPF	/	UPF were classified using NOVA system as industrial formulations composed of few or no whole foods and containing additives, preservatives, and chemically modified ingredients. These were grouped into nine categories, such as ultra-processed breads and breakfast foods; packaged sweet snacks and desserts; packaged savory snacks; sugar- and artificially sweetened beverages; ready-to-eat or ready-to-heat mixed dishes; meat-, poultry-, or seafood-based ready-to-eat products; sauces, spreads, and condiments; dairy-based desserts; other ultra-processed foods[12].

DASH, Dietary Approaches to Stop Hypertension; EDIH, empirical dietary index for hyperinsulinemia; EDIP, empirical dietary inflammation pattern; HLCD, healthy low-carbohydrate diet; hPDI, healthy plant-based diet index; LCD, low-carbohydrate diet; MedDiet, Mediterranean diet; MET, metabolic equivalent; PDI, plant-based diet index; PHDI, Planetary Health Diet Index; ULCD, unhealthy low-carbohydrate diet; uPDI, unhealthy plant-based diet index; UPF, ultra-processed food

© 2026 Xia T et al. JAMA Network Open.

eTable 3. Calculation of the diet scores

Component	PDI	hPDI	uPDI	DASH	MedDiet	PHDI	LCD	HLCD	ULCD	EDIP	EDIH	UPF
Fruits	Q1-Q5: 1-5 points	Q1-Q5: 1-5 points	Q1-Q5: 5-1 points	Q1-Q5: 1-5 points	0/1 (≥ median = 1)	0–10 points (≥200 g/day = max score)					β × servings/day	
Tomatoes										β × servings/day	β × servings/day	
Vegetables	Q1-Q5: 1-5 points	Q1-Q5: 1-5 points	Q1-Q5: 5-1 points	Q1-Q5: 1-5 points	0/1 (≥ median = 1)	0–10 points (≥300 g/day = max score)						
Starchy vegetables						0–10 points (0 if ≥200 g/day; 10 if ≤50 g/day)						
Leafy green vegetables										β × servings/day	β × servings/day	
Dark yellow vegetables										β × servings/day		
Other vegetables										β × servings/day		
Potatoes	Q1-Q5: 1-5 points	Q1-Q5: 5-1 points	Q1-Q5: 1-5 points									
Whole grains	Q1-Q5: 1-5 points	Q1-Q5: 1-5 points	Q1-Q5: 5-1 points	Q1-Q5: 1-5 points	0/1 (≥ median = 1)	0–10 points (≥75 g/day women; ≥90 g/day men = max score)						
Refined grains	Q1-Q5: 1-5 points	Q1-Q5: 5-1 points	Q1-Q5: 1-5 points							β × servings/day		
Nuts and legumes				Q1-Q5: 1-5 points								
Nuts	Q1-Q5: 1-5 points	Q1-Q5: 1-5 points	Q1-Q5: 5-1 points		0/1 (≥ median = 1)	0–10 points (≥50 g/day = max score)						
Legumes	Q1-Q5: 1-5 points	Q1-Q5: 1-5 points	Q1-Q5: 5-1 points		0/1 (≥ median = 1)	0–10 points (≥100 g/day = max score; weight 0.5)						
Soy						0–10 points (≥50 g/day = max score; weight 0.5)						
Red, processed meat and poultry	Q1-Q5: 5-1 points	Q1-Q5: 5-1 points	Q1-Q5: 5-1 points									
Red and Processed meats				Q1-Q5: 5-1 points	0/1 (< median = 1)	0–10 points (0 if ≥100 g/day; 10 if ≤14 g/day)						
Red meats										β × servings/day	β × servings/day	
Processed meats										β × servings/day	β × servings/day	
Organ meats										β × servings/day		
Poultry						0–10 points (0 if ≥100 g/day; 10 if ≤29 g/day)					β × servings/day	
Eggs	Q1-Q5: 5-1 points	Q1-Q5: 5-1 points	Q1-Q5: 5-1 points			0–10 points (0 if ≥120 g/day; 10 if ≤13 g/day)					β × servings/day	
Fish and seafood	Q1-Q5: 5-1 points	Q1-Q5: 5-1 points	Q1-Q5: 5-1 points		0/1 (≥ median = 1)	0–10 points (≥28 g/day = max score)						
Other fish										β × servings/day	β × servings/day	
Miscellaneous animal-based foods	Q1-Q5: 5-1 points	Q1-Q5: 5-1 points	Q1-Q5: 5-1 points									

© 2026 Xia T et al. JAMA Network Open.

Sweets and desserts	Q1-Q5: 1-5 points	Q1-Q5: 5-1 points	Q1-Q5: 1-5 points								
French fries											β × servings/day
Snacks										β × servings/day	
Pizza										β × servings/day	
Animal fat	Q1-Q5: 5-1 points	Q1-Q5: 5-1 points	Q1-Q5: 5-1 points								
Vegetable oils	Q1-Q5: 1-5 points	Q1-Q5: 1-5 points	Q1-Q5: 5-1 points								
Creamy soups											β × servings/day
Margarine											β × servings/day
Butter											β × servings/day
High-energy beverages	Q1-Q5: 1-5 points	Q1-Q5: 5-1 points	Q1-Q5: 1-5 points	Q1-Q5: 5-1 points						β × servings/day	β × servings/day
Low-energy beverages										β × servings/day	β × servings/day
Fruit juices	Q1-Q5: 1-5 points	Q1-Q5: 5-1 points	Q1-Q5: 1-5 points							β × servings/day	
Total alcohol					0/1 (moderate range = 1)						
Wine										β × servings/day	β × servings/day
Beer										β × servings/day	
Tea and coffee	Q1-Q5: 1-5 points	Q1-Q5: 1-5 points	Q1-Q5: 5-1 points								
Tea										β × servings/day	
Coffee										β × servings/day	β × servings/day
Total dairy	Q1-Q5: 5-1 points	Q1-Q5: 5-1 points	Q1-Q5: 5-1 points			0–10 points (0 if ≥1000 g/day; 10 if ≤250 g/day)					
High-fat dairy											β × servings/day
Low-fat dairy				Q1-Q5: 1-5 points							β × servings/day
Polyunsaturated fatty acids							D1-D10: 0-10 points	D1-D10: 0-10 points			
Plant Monounsaturated Fat							D1-D10: 0-10 points	D1-D10: 0-10 points			
Animal Monounsaturated Fat							D1-D10: 0-10 points		D1-D10: 0-10 points		
Ratio of monounsaturated to saturated fatty acids					0/1 (≥ median = 1)						

© 2026 Xia T et al. JAMA Network Open.

Added unsaturated fats		0–10 points (0 if ≤3.5% TEI; 10 if ≥21% TEI)				
Added saturated fats and trans fats		0–10 points (0 if ≥10% TEI; 10 if 0% TEI)	D1-D10: 0-10 points		D1-D10: 0-10 points	
Added sugar and fruit juices		0–10 points (0 if ≥25% TEI; 10 if ≤5% TEI)				
Sodium	Q1-Q5: 5-1 points					
Fruits carbohydrate			D1-D10: 10-0 points		D1-D10: 10-0 points	
Vegetables carbohydrate			D1-D10: 10-0 points		D1-D10: 10-0 points	
Potatoes carbohydrate			D1-D10: 10-0 points	D1-D10: 10-0 points		
Whole grains carbohydrate			D1-D10: 10-0 points		D1-D10: 10-0 points	
Refined grains carbohydrate			D1-D10: 10-0 points	D1-D10: 10-0 points		
Legume carbohydrate			D1-D10: 10-0 points		D1-D10: 10-0 points	
Added sugar and fruit juices carbohydrate			D1-D10: 10-0 points	D1-D10: 10-0 points		
Animal protein			D1-D10: 0-10 points		D1-D10: 0-10 points	
Vegetable protein			D1-D10: 0-10 points	D1-D10: 0-10 points		
Ultra-processed breads and breakfast foods						Servings/day summed
Packaged sweet snacks and desserts						Servings/day summed
Packaged savory snacks						Servings/day summed
Sugar- and artificially sweetened beverages						Servings/day summed
Ready-to-eat or ready-to-heat mixed dishes						Servings/day summed
Meat-, poultry-, or seafood-based ready-to-eat products						Servings/day summed
Sauces, spreads, and condiments						Servings/day summed
Dairy-based desserts						Servings/day summed
Other ultra-processed foods						Servings/day summed

© 2026 Xia T et al. JAMA Network Open.

Q1–Q5: Quintiles of intake.
D1–D10: Deciles of intake.
β × servings/day: Food group intake (servings/day) multiplied by the published regression coefficient (β) derived from reduced rank regression models predicting inflammatory or insulinemic biomarkers.
Servings/day summed: Total daily intake of all food items classified as ultra-processed according to the NOVA classification system, summed across FFQ items as servings/day.
TEI: Total Energy Intake.
DASH, Dietary Approaches to Stop Hypertension; EDIH, empirical dietary index for hyperinsulinemia; EDIP, empirical dietary inflammation pattern; HLCD, healthy low-carbohydrate diet; hPDI, healthy plant-based diet index; LCD, low-carbohydrate diet; MedDiet, Mediterranean diet; PDI, plant-based diet index; PHDI, Planetary Health Diet Index; ULCD, unhealthy low-carbohydrate diet; uPDI, unhealthy plant-based diet index; UPF, ultra-processed food

© 2026 Xia T et al. JAMA Network Open.

eTable 4. Characteristics of study population in the lowest and highest quintiles of dietary patterns

**Characteristics by diet pattern**	Quintile 1, No. (%) or Mean (SD)	Quintile 5, No. (%) or Mean (SD)
**PDI**		
Diet score	45.19 (2.69)	61.90 (2.80)
Age (years)	45.97 (2.98)	45.40 (2.91)
Race/ethnicity, No. (%)		
White	7309 (95.4)	7087 (94.2)
Asian; American Indian/Alaska Native; Native Hawaiian/Other Pacific Islander	173 (2.3)	188 (2.5)
Black	69 (0.9)	98 (1.3)
Hispanic	112 (1.5)	154 (2.1)
Married, No. (%)	6063 (79.1)	6253 (83.1)
Income, No. (%)		
<50k	812 (10.6)	872 (11.6)
50k–99k	2609 (34.1)	2613 (34.7)
≥100k	2199 (28.7)	2124 (28.22)
Missing	2043 (28.7)	1918 (25.5)
Nulliparity, No. (%)	1440 (18.8)	1289 (17.1)
Postmenopausal hormone use, No. (%)		
Never	2855 (37.3)	2478 (32.9)
Ever	2725 (35.6)	3304 (43.9)
Missing	2083 (27.2)	1745 (23.2)
Alcohol intake (g/d)	5.99 (9.95)	4.72 (7.49)
Total energy (kcal/d)	1501.43 (471.97)	2130.65 (535.21)
Physical activity (MET-hr/week)	19.04 (24.12)	25.37 (27.17)
Never smoke, No. (%)	4777 (62.3)	4987 (66.3)
Weight (kg)	70.37 (13.95)	65.28 (11.97)
Body mass index (kg/m²)	25.99 (4.76)	24.01 (4.04)
**hPDI**		
Diet score	44.81 (3.19)	64.42 (3.17)
Age (years)	45.72 (2.99)	45.64 (2.97)
Race/ethnicity, No. (%)		
White	7419 (96.2)	7117 (93.4)
Asian; American Indian/Alaska Native; Native Hawaiian/Other Pacific Islander	135 (1.8)	229 (3.0)
Black	76 (1.0)	89 (1.2)
Hispanic	84 (1.1)	187 (2.5)
Married, No. (%)	6429 (83.3)	5998 (78.7)
Income, No. (%)		
<50k	1045 (13.6)	726 (9.5)

© 2026 Xia T et al. JAMA Network Open.

50k–99k	2929 (38.0)	2460 (32.3)
≥100k	1755 (22.8)	2477 (32.5)
Missing	1985 (25.7)	1959 (25.7)
Nulliparity, No. (%)	1237 (16.0)	1612 (21.2)
Postmenopausal hormone use, No. (%)		
Never	2988 (38.7)	2443 (32.1)
Ever	2553 (33.1)	3415 (44.8)
Missing	2173 (28.2)	1764 (23.1)
Alcohol intake (g/d)	4.05 (7.35)	5.45 (8.75)
Total energy (kcal/d)	2059.68 (545.36)	1616.54 (503.26)
Physical activity (MET-hr/week)	17.03 (22.68)	28.26 (30.70)
Never smoke, No. (%)	5449 (70.6)	4711 (61.8)
Weight (kg)	70.74 (14.49)	65.02 (11.45)
Body mass index (kg/m²)	26.04 (4.98)	24.01 (3.84)
**uPDI**		
Diet score	42.74 (3.13)	63.67 (3.31)
Age (years)	46.08 (2.94)	45.30 (2.99)
Race/ethnicity, No. (%)		
White	7284 (95.8)	7179 (94.4)
Asian; American Indian/Alaska Native; Native Hawaiian/Other Pacific Islander	142 (1.9)	173 (2.3)
Black	40 (0.5)	152 (2.0)
Hispanic	136 (1.8)	102 (1.3)
Married, No. (%)	6297 (82.8)	6092 (80.1)
Income, No. (%)		
<50k	652 (8.6)	1024 (13.5)
50k–99k	2502 (32.9)	2783 (36.6)
≥100k	2649 (34.9)	1667 (21.9)
Missing	1799 (23.7)	2132 (28.0)
Nulliparity, No. (%)	1306 (17.2)	1349 (17.7)
Postmenopausal hormone use, No. (%)		
Never	2657 (35.0)	2738 (36.0)
Ever	3182 (41.9)	2772 (36.4)
Missing	1763 (23.2)	2096 (27.6)
Alcohol intake (g/d)	7.36 (9.83)	2.66 (6.03)
Total energy (kcal/d)	2072.20 (529.77)	1572.96 (502.22)
Physical activity (MET-hr/week)	27.72 (30.49)	16.02 (21.19)
Never smoke, No. (%)	4572 (60.1)	5360 (70.5)
Weight (kg)	68.39 (12.86)	68.13 (13.72)
Body mass index (kg/m²)	25.09 (4.35)	25.25 (4.73)
**DASH**		

© 2026 Xia T et al. JAMA Network Open.

Diet score	17.33 (1.86)	30.57 (1.84)
Age (years)	45.39 (3.00)	45.87 (2.95)
Race/ethnicity, No. (%)		
White	7198 (94.9)	6948 (94.9)
Asian; American Indian/Alaska Native; Native Hawaiian/Other Pacific Islander	188 (2.5)	146 (2.0)
Black	102 (1.3)	74 (1.0)
Hispanic	101 (1.3)	152 (2.1)
Married, No. (%)	6153 (81.1)	5922 (80.9)
Income, No. (%)		
<50k	973 (12.8)	733 (10.0)
50k–99k	2817 (37.1)	2399 (32.8)
≥100k	1736 (22.9)	2407 (32.9)
Missing	2063 (27.2)	1781 (24.3)
Nulliparity, No. (%)	1285 (16.9)	1422 (19.4)
Postmenopausal hormone use, No. (%)		
Never	2812 (37.1)	2516 (34.4)
Ever	2671 (35.2)	3168 (43.3)
Missing	2106 (27.8)	1636 (22.4)
Alcohol intake (g/d)	4.39 (8.44)	5.64 (8.56)
Total energy (kcal/d)	1585.48 (524.70)	2060.60 (521.01)
Physical activity (MET-hr/week)	14.80 (20.85)	30.73 (31.98)
Never smoke, No. (%)	4699 (61.9)	4854 (66.3)
Weight (kg)	70.44 (14.34)	65.37 (11.66)
Body mass index (kg/m²)	26.11 (4.92)	23.95 (3.95)
**MedDiet**		
Diet score	2.24 (0.71)	7.10 (0.60)
Age (years)	45.50 (2.99)	45.88 (2.94)
Race/ethnicity, No. (%)		
White	8179 (95.7)	7157 (94.3)
Asian; American Indian/Alaska Native; Native Hawaiian/Other Pacific Islander	171 (2.0)	169 (2.2)
Black	91 (1.1)	93 (1.2)
Hispanic	103 (1.2)	170 (2.2)
Married, No. (%)	6948 (81.3)	6219 (82.0)
Income, No. (%)		
<50k	1116 (13.1)	677 (8.9)
50k–99k	3165 (37.0)	2446 (32.2)
≥100k	1875 (22.0)	2674 (35.2)
Missing	2388 (28.0)	1792 (23.6)
Nulliparity, No. (%)	1397 (16.4)	1403 (18.5)

© 2026 Xia T et al. JAMA Network Open.

Postmenopausal hormone use, No. (%)		
Never	3239 (37.9)	2582 (34.0)
Ever	2906 (34.0)	3270 (43.1)
Missing	2399 (28.1)	1737 (22.9)
Alcohol intake (g/d)	3.53 (8.08)	6.66 (8.05)
Total energy (kcal/d)	1579.66 (512.33)	2071.69 (522.58)
Physical activity (MET-hr/week)	14.84 (20.69)	29.46 (30.59)
Never smoke, No. (%)	5558 (65.1)	4812 (63.4)
Weight (kg)	70.29 (14.32)	65.94 (11.82)
Body mass index (kg/m²)	25.99 (4.91)	24.21 (4.00)
**PHDI**		
Diet score	66.32 (5.34)	100.84 (6.09)
Age (years)	44.75 (2.95)	46.50 (2.82)
Race/ethnicity, No. (%)		
White	7388(96.5)	7089 (92.6)
Asian; American Indian/Alaska Native; Native Hawaiian/Other Pacific Islander	99 (1.3)	287 (3.8)
Black	89 (1.2)	102 (1.3)
Hispanic	80 (1.0)	178 (2.3)
Married, No. (%)	6227 (81.3)	6178 (80.7)
Income, No. (%)		
<50k	1093 (14.3)	691 (9.0)
50k–99k	2810 (36.7)	2466 (32.2)
≥100k	1597 (20.9)	2636 (34.4)
Missing	2156 (28.2)	1863 (24.3)
Nulliparity, No. (%)	1232 (16.1)	1569 (20.5)
Postmenopausal hormone use, No. (%)		
Never	2448 (32.0)	2934 (38.3)
Ever	3227 (42.2)	2901 (37.9)
Missing	1981 (25.9)	1821 (23.8)
Alcohol intake (g/d)	3.54 (7.63)	6.38 (9.04)
Total energy (kcal/d)	1871.41 (578.25)	1811.25 (530.06)
Physical activity (MET-hr/week)	15.25 (20.41)	30.39 (31.64)
Never smoke, No. (%)	4876 (63.7)	5058 (66.1)
Weight (kg)	69.93 (14.41)	65.41 (11.71)
Body mass index (kg/m²)	25.81 (4.95)	24.09 (3.96)
**LCD**		
Diet score	6.46 (2.36)	23.20 (2.32)
Age (years)	45.59 (3.00)	45.65 (3.01)
Race/ethnicity, No. (%)		
White	6986 (92.9)	7038 (95.6)

© 2026 Xia T et al. JAMA Network Open.

Asian; American Indian/Alaska Native; Native Hawaiian/Other Pacific Islander	253 (3.4)	143 (1.9)
Black	152 (2.0)	61 (0.8)
Hispanic	130 (1.7)	119 (1.6)
Married, No. (%)	5916 (78.7)	6052 (82.2)
Income, No. (%)		
<50k	968 (12.9)	739 (10.0)
50k–99k	2760 (36.7)	2406 (32.7)
≥100k	1800 (23.9)	2317 (31.5)
Missing	1993 (26.5)	1899 (25.8)
Nulliparity, No. (%)	1522 (20.2)	1226 (16.7)
Postmenopausal hormone use, No. (%)		
Never	2711 (36.1)	2375 (32.3)
Ever	2854 (38.0)	3159 (42.9)
Missing	1956 (26.0)	1827 (24.8)
Alcohol intake (g/d)	3.39 (6.60)	5.37 (8.11)
Total energy (kcal/d)	1839.22 (552.36)	1710.85 (545.20)
Physical activity (MET-hr/week)	24.35 (29.43)	19.53 (23.43)
Never smoke, No. (%)	5245 (69.7)	4440 (60.3)
Weight (kg)	65.42 (12.26)	70.47 (13.88)
Body mass index (kg/m²)	24.12 (4.17)	26.01 (4.74)
**HLCD**		
Diet score	7.76 (2.43)	23.05 (2.21)
Age (years)	45.61 (2.99)	45.98 (2.96)
Race/ethnicity, No. (%)		
White	7166 (95.1)	7148 (94.0)
Asian; American Indian/Alaska Native; Native Hawaiian/Other Pacific Islander	177 (2.4)	191 (2.5)
Black	118 (1.6)	82 (1.1)
Hispanic	78 (1.0)	182 (2.4)
Married, No. (%)	6196 (82.2)	6125 (80.6)
Income, No. (%)		
<50k	1031 (13.7)	722 (9.5)
50k–99k	2869 (38.1)	2405 (31.6)
≥100k	1607 (21.3)	2600 (34.2)
Missing	2032 (27.0)	1876 (24.7)
Nulliparity, No. (%)	1253 (16.6)	1503 (19.8)
Postmenopausal hormone use, No. (%)		
Never	2934 (38.9)	2559 (33.7)
Ever	2507 (33.3)	3334 (43.9)
Missing	2098 (27.8)	1710 (22.5)

© 2026 Xia T et al. JAMA Network Open.

Alcohol intake (g/d)	3.69 (7.44)	5.93 (8.46)
Total energy (kcal/d)	1843.94 (558.49)	1802.81 (547.26)
Physical activity (MET-hr/week)	16.78 (22.51)	27.85 (30.01)
Never smoke, No. (%)	5076 (67.3)	4788 (63.0)
Weight (kg)	69.13 (13.99)	66.05 (11.94)
Body mass index (kg/m²)	25.54 (4.77)	24.30 (4.07)
**ULCD**		
Diet score	5.83 (2.50)	22.86 (2.33)
Age (years)	45.74 (2.95)	45.51 (3.00)
Race/ethnicity, No. (%)		
White	7193 (92.2)	7504 (96.1)
Asian; American Indian/Alaska Native; Native Hawaiian/Other Pacific Islander	270 (3.5)	152 (2.0)
Black	142 (1.8)	62 (0.8)
Hispanic	197 (2.5)	93 (1.2)
Married, No. (%)	6117 (78.4)	6398 (81.9)
Income, No. (%)		
<50k	844 (10.8)	952 (12.2)
50k–99k	2654 (34.0)	2804 (35.9)
≥100k	2314 (30.0)	1990 (25.5)
Missing	1990 (25.5)	2065 (26.4)
Nulliparity, No. (%)	1744 (22.4)	1248 (16.0)
Postmenopausal hormone use, No. (%)		
Never	2688 (34.5)	2771 (35.5)
Ever	3236 (41.5)	2951 (37.8)
Missing	1878 (24.1)	2089 (26.7)
Alcohol intake (g/d)	5.29 (8.73)	4.44 (7.73)
Total energy (kcal/d)	1818.75 (549.04)	1753.83 (562.46)
Physical activity (MET-hr/week)	29.29 (32.20)	15.99 (21.02)
Never smoke, No. (%)	5177 (66.4)	4818 (61.7)
Weight (kg)	64.28 (11.21)	71.47 (14.36)
Body mass index (kg/m²)	23.68 (3.79)	26.36 (4.92)
**EDIP**		
Diet score	-0.53 (0.19)	0.30 (0.18)
Age (years)	45.87 (2.93)	45.37 (2.97)
Race/ethnicity, No. (%)		
White	7483 (97.7)	7011 (91.6)
Asian; American Indian/Alaska Native; Native Hawaiian/Other Pacific Islander	64 (0.8)	365 (4.8)
Black	24 (0.3)	130 (1.7)
Hispanic	85 (1.1)	150 (2.0)

© 2026 Xia T et al. JAMA Network Open.

Married, No. (%)	6270 (81.9)	6143 (80.2)
Income, No. (%)		
<50k	705 (9.2)	1008 (13.2)
50k–99k	2517 (32.9)	2812 (36.7)
≥100k	2655 (34.7)	1756 (22.9)
Missing	1779 (23.3)	2080 (27.2)
Nulliparity, No. (%)	1380 (18.0)	1436 (18.8)
Postmenopausal hormone use, No. (%)		
Never	2742 (35.8)	2684 (35.1)
Ever	3064 (40.0)	2941 (38.4)
Missing	1850 (24.2)	2031 (26.5)
Alcohol intake (g/d)	11.46 (13.11)	1.96 (4.77)
Total energy (kcal/d)	1851.70 (542.92)	1909.73 (591.12)
Physical activity (MET-hr/week)	25.85 (29.94)	18.14 (24.08)
Never smoke, No. (%)	4179 (54.6)	5515 (72.0)
Weight (kg)	65.47 (11.37)	71.69 (14.87)
Body mass index (kg/m²)	24.03 (3.84)	26.55 (5.04)
**EDIH**		
Diet score	-0.02 (0.11)	0.64 (0.17)
Age (years)	46.06 (2.90)	45.28 (2.98)
Race/ethnicity, No. (%)		
White	7344 (95.9)	7251 (94.7)
Asian; American Indian/Alaska Native; Native Hawaiian/Other Pacific Islander	140 (1.8)	189 (2.5)
Black	56 (0.7)	96 (1.3)
Hispanic	116 (1.5)	120 (1.6)
Married, No. (%)	6049 (79.0)	6282 (82.1)
Income, No. (%)		
<50k	686 (9.0)	1061 (13.9)
50k–99k	2430 (31.7)	2934 (38.3)
≥100k	2686 (35.1)	1671 (21.8)
Missing	1854 (24.2)	1990 (26.0)
Nulliparity, No. (%)	1650 (21.6)	1279 (16.7)
Postmenopausal hormone use, No. (%)		
Never	2768 (36.2)	2638 (34.5)
Ever	3058 (39.9)	3034 (39.6)
Missing	1830 (23.9)	1984 (25.9)
Alcohol intake (g/d)	9.84 (12.39)	3.16 (6.96)
Total energy (kcal/d)	1646.29 (513.72)	2126.62 (568.67)
Physical activity (MET-hr/week)	27.53 (30.91)	17.71 (23.34)
Never smoke, No. (%)	4415 (57.7)	5260 (68.7)

© 2026 Xia T et al. JAMA Network Open.

Weight (kg)	64.00 (10.68)	72.67 (14.93)
Body mass index (kg/m²)	23.54 (3.58)	26.79 (5.08)
**UPF**		
Diet score	1.16 (1.37)	10.61 (2.10)
Age (years)	45.70 (2.95)	45.48 (2.97)
Race/ethnicity, No. (%)		
White	6993 (91.4)	7427 (97.0)
Asian; American Indian/Alaska Native; Native Hawaiian/Other Pacific Islander	352 (4.6)	80 (1.1)
Black	144 (1.9)	49 (0.6)
Hispanic	162 (2.1)	99 (1.3)
Married, No. (%)	6069 (79.3)	6213 (81.2)
Income, No. (%)		
<50k	841 (11.0)	907 (11.9)
50k–99k	2420 (31.6)	2896 (37.8)
≥100k	2012 (26.3)	2090 (27.3)
Missing	2378 (31.1)	1762 (23.0)
Nulliparity, No. (%)	1429 (18.7)	1419 (18.5)
Postmenopausal hormone use, No. (%)		
Never	2749 (35.9)	2505 (32.7)
Ever	2797 (36.6)	3297 (43.1)
Missing	2105 (27.5)	1853 (24.2)
Alcohol intake (g/d)	5.35 (8.77)	4.54 (8.31)
Total energy (kcal/d)	1596.35 (572.69)	2154.95 (557.52)
Physical activity (MET-hr/week)	24.24 (29.65)	19.98 (24.20)
Never smoke, No. (%)	4892 (63.9)	4798 (62.7)
Weight (kg)	66.53 (12.83)	71.34 (14.37)
Body mass index (kg/m²)	24.63 (4.32)	26.17 (4.92)

Data are presented as mean (standard deviation, SD) for continuous variables and number (percentage), No. (%), for categorical variables.

DASH, Dietary Approaches to Stop Hypertension; EDIH, empirical dietary index for hyperinsulinemia; EDIP, empirical dietary inflammation pattern; HLCD, healthy low-carbohydrate diet; hPDI, healthy plant-based diet index; LCD, low-carbohydrate diet; MedDiet, Mediterranean diet; MET, metabolic equivalent of task; PDI, plant-based diet index; PHDI, Planetary Health Diet Index; ULCD, unhealthy low-carbohydrate diet; uPDI, unhealthy plant-based diet index; UPF, ultra-processed food.

© 2026 Xia T et al. JAMA Network Open.

eTable 5. Associations [β (95% CI)] of dietary patterns (Quintile 5 vs. 1) with weight change during menopause in the Nurse Health Study II

	Estimate	95% CI	P-value
PDI			
Age and race model	-0.29	(-0.31, -0.26)	<0.001*
Multivariable model	-0.30	(-0.32, -0.27)	<0.001*
Multivariable + BMI model	-0.23	(-0.26, -0.21)	<0.001*
hPDI			
Age and race model	-0.15	(-0.17, -0.13)	<0.001*
Multivariable model	-0.11	(-0.14, -0.09)	<0.001*
Multivariable + BMI model	-0.08	(-0.10, -0.05)	<0.001*
uPDI			
Age and race model	0.06	(0.04, 0.08)	<0.001*
Multivariable model	-0.04	(-0.06, -0.02)	0.001*
Multivariable + BMI model	-0.02	(-0.04, -0.0003)	0.05
DASH			
Age and race model	-0.30	(-0.32, -0.28)	<0.001*
Multivariable model	-0.24	(-0.27, -0.22)	<0.001*
Multivariable + BMI model	-0.19	(-0.22, -0.17)	<0.001*
MedDiet			
Age and race model	-0.25	(-0.27, -0.23)	<0.001*
Multivariable model	-0.18	(-0.21, -0.16)	<0.001*
Multivariable + BMI model	-0.15	(-0.17, -0.13)	<0.001*
PHDI			
Age and race model	-0.28	(-0.31, -0.26)	<0.001*
Multivariable model	-0.20	(-0.23, -0.18)	<0.001*
Multivariable + BMI model	-0.17	(-0.19, -0.15)	<0.001*
LCD			
Age and race model	0.24	(0.22, 0.26)	<0.001*
Multivariable model	0.24	(0.22, 0.26)	<0.001*
Multivariable + BMI model	0.18	(0.16, 0.20)	<0.001*
HLCD			
Age and race model	-0.14	(-0.16, -0.12)	<0.001*
Multivariable model	-0.07	(-0.10, -0.05)	<0.001*
Multivariable + BMI model	-0.06	(-0.08, -0.03)	<0.001*

© 2026 Xia T et al. JAMA Network Open.

ULCD			
Age and race model	0.32	(0.30, 0.34)	<0.001*
Multivariable model	0.27	(0.25, 0.29)	<0.001*
Multivariable + BMI model	0.21	(0.19, 0.23)	<0.001*
EDIP			
Age and race model	0.29	(0.27, 0.31)	<0.001*
Multivariable model	0.26	(0.24, 0.28)	<0.001*
Multivariable + BMI model	0.20	(0.18, 0.22)	<0.001*
EDIH			
Age and race model	0.36	(0.34, 0.38)	<0.001*
Multivariable model	0.36	(0.34, 0.38)	<0.001*
Multivariable + BMI model	0.28	(0.26, 0.30)	<0.001*
UPF			
Age and race model	0.14	(0.11, 0.16)	<0.001*
Multivariable model	0.15	(0.12, 0.17)	<0.001*
Multivariable + BMI model	0.11	(0.08, 0.13)	<0.001*

Adjusted mean annualized weight change (kg/year) and 95%CI were reported, which were estimated using generalized estimating equations with repeated measures to account for within-person correlation across follow-up intervals, specifying an unstructured working correlation matrix. *indicated Bonferroni-adjusted P < 0.05. Sample size reflects the number of participants contributing at least one interval to the Generalized Estimating Equations analysis (n=48,317). Because participants could enter or exit at different periods depending on interval-specific exclusions (e.g., incident disease, implausible/missing weight change), the analytic sample size is larger than the baseline sample shown in Table 1.
Age and race model: adjust for baseline age (≤45, >45 years), race (White; Asian, American Indian/Alaska Native, Native Hawaiian/Other; Black; Hispanic). Multivariable model: adjust for baseline age (≤45, >45 years), race (White; Asian, American Indian/Alaska Native, Native Hawaiian/Other; Black; Hispanic), marital status (never, ever), income (<50k, 50k–99k, ≥100k, missing), postmenopausal hormone use (never, ever, missing), parity (nulliparous, 1 and 2, or ≥3 births), smoking status (never, ever), alcohol (g/day; excluding EDIP, EDIH, and MedDiet scores, where alcohol is included in the scoring criteria), total energy intake (<median, ≥median), and physical activity (<1000, ≥1000 MET-mins/week).
Multivariable and BMI model: adjust for baseline age (≤45, >45 years), race (White; Asian, American Indian/Alaska Native, Native Hawaiian/Other; Black; Hispanic), marital status (never, ever), income (<50k, 50k–99k, ≥100k, missing), postmenopausal hormone use (never, ever, missing), parity (nulliparous, 1 and 2, or ≥3 births), smoking status (never, ever), alcohol (g/day), total energy intake (<median, ≥median), physical activity (<1000, ≥1000 MET-mins/week), and each interval baseline body mass index (>18 and <25 kg/m2, ≥25 kg/m2).
BMI: body mass index; DASH, Dietary Approaches to Stop Hypertension; EDIH, empirical dietary index for hyperinsulinemia; EDIP, empirical dietary inflammation pattern; HLCD, healthy low-carbohydrate diet; hPDI, healthy plant-based diet index; LCD, low-carbohydrate diet; MedDiet, Mediterranean diet; MET, metabolic equivalent; PDI, plant-based diet index; PHDI, Planetary Health Diet Index; ULCD, unhealthy low-carbohydrate diet; uPDI, unhealthy plant-based diet index; UPF, ultra-processed food

© 2026 Xia T et al. JAMA Network Open.

eTable 6. Associations [β (95% CI)] of dietary patterns (per SD) with weight change during menopause in the Nurse Health Study II

	HR	95% CI	P-value
PDI			
Age and race model	-0.103	(-0.110, -0.096)	<0.001*
Multivariable model	-0.108	(-0.116, -0.101)	<0.001*
Multivariable + BMI model	-0.086	(-0.093, -0.079)	<0.001*
hPDI			
Age and race model	-0.052	(-0.058, -0.045)	<0.001*
Multivariable model	-0.040	(-0.047, -0.033)	<0.001*
Multivariable + BMI model	-0.026	(-0.033, -0.019)	<0.001*
uPDI			
Age and race model	0.017	(0.010, 0.024)	<0.001*
Multivariable model	-0.020	(-0.027, -0.012)	<0.001*
Multivariable + BMI model	-0.013	(-0.020, -0.005)	0.001*
DASH			
Age and race model	-0.103	(-0.110, -0.096)	<0.001*
Multivariable model	-0.084	(-0.091, -0.076)	<0.001*
Multivariable + BMI model	-0.066	(-0.073, -0.059)	<0.001*
MedDiet			
Age and race model	-0.088	(-0.095, -0.081)	<0.001*
Multivariable model	-0.065	(-0.073, -0.058)	<0.001*
Multivariable + BMI model	-0.051	(-0.059, -0.044)	<0.001*
PHDI			
Age and race model	-0.103	(-0.110, -0.096)	<0.001*
Multivariable model	-0.074	(-0.082, -0.067)	<0.001*
Multivariable + BMI model	-0.061	(-0.068, -0.055)	<0.001*
LCD			
Age and race model	0.083	(0.075, 0.089)	<0.001*
Multivariable model	0.083	(0.076, 0.089)	<0.001*
Multivariable + BMI model	0.063	(0.057, 0.070)	<0.001*
HLCD			
Age and race model	-0.050	(-0.057, -0.043)	<0.001*
Multivariable model	-0.027	(-0.034, -0.020)	<0.001*
Multivariable + BMI model	-0.021	(-0.027, -0.014)	<0.001*

© 2026 Xia T et al. JAMA Network Open.

ULCD			
Age and race model	0.113	(0.106, 0.120)	<0.001*
Multivariable model	0.095	(0.088, 0.102)	<0.001*
Multivariable + BMI model	0.073	(0.066, 0.079)	<0.001*
EDIP			
Age and race model	0.097	(0.090, 0.104)	<0.001*
Multivariable model	0.086	(0.078, 0.093)	<0.001*
Multivariable + BMI model	0.066	(0.059, 0.073)	<0.001*
EDIH			
Age and race model	0.126	(0.119, 0.133)	<0.001*
Multivariable model	0.128	(0.121, 0.136)	<0.001*
Multivariable + BMI model	0.101	(0.093, 0.108)	<0.001*
UPF			
Age and race model	0.049	(0.042, 0.056)	<0.001*
Multivariable model	0.049	(0.041, 0.057)	<0.001*
Multivariable + BMI model	0.036	(0.029, 0.043)	<0.001*

Adjusted mean annualized weight change (kg/year) and 95%CI were reported, which were estimated using generalized estimating equations with repeated measures to account for within-person correlation across follow-up intervals, specifying an unstructured working correlation matrix. * indicated Bonferroni-adjusted P < 0.05. Sample size reflects the number of participants contributing at least one interval to the Generalized Estimating Equations analysis (n=48,317). Because participants could enter or exit at different periods depending on interval-specific exclusions (e.g., incident disease, implausible/missing weight change), the analytic sample size is larger than the baseline sample shown in Table 1.

Age and race model: adjust for baseline age (≤45, >45 years), race (White; Asian, American Indian/Alaska Native, Native Hawaiian/Other; Black; Hispanic). Multivariable model: adjust for baseline age (≤45, >45 years), race (White; Asian, American Indian/Alaska Native, Native Hawaiian/Other; Black; Hispanic), marital status (never, ever), income (<50k, 50k–99k, ≥100k, missing), postmenopausal hormone use (never, ever, missing), parity (nulliparous, 1 and 2, or ≥3 births), smoking status (never, ever), alcohol (g/day; excluding EDIP, EDIH, and MedDiet scores, where alcohol is included in the scoring criteria), total energy intake (<median, ≥median), and physical activity (<1000, ≥1000 MET-mins/week). Multivariable and BMI model: adjust for baseline age (≤45, >45 years), race (White; Asian, American Indian/Alaska Native, Native Hawaiian/Other; Black; Hispanic), marital status (never, ever), income (<50k, 50k–99k, ≥100k, missing), postmenopausal hormone use (never, ever, missing), parity (nulliparous, 1 and 2, or ≥3 births), smoking status (never, ever), alcohol (g/day), total energy intake (<median, ≥median), physical activity (<1000, ≥1000 MET-mins/week), and each interval baseline body mass index (>18 and <25 kg/m2, ≥25 kg/m2).

BMI: body mass index; DASH, Dietary Approaches to Stop Hypertension; EDIH, empirical dietary index for hyperinsulinemia; EDIP, empirical dietary inflammation pattern; HLCD, healthy low-carbohydrate diet; hPDI, healthy plant-based diet index; LCD, low-carbohydrate diet; MedDiet, Mediterranean diet; MET, metabolic equivalent; PDI, plant-based diet index; PHDI, Planetary Health Diet Index; ULCD, unhealthy low-carbohydrate diet; uPDI, unhealthy plant-based diet index; UPF, ultra-processed food

© 2026 Xia T et al. JAMA Network Open.

eTable 7. Proportion of women had weight loss in diet quintile 5 vs 1

Dietary Pattern	Quintile 1 of diet % with Weight Loss	Quintile 5 % with Weight Loss	P-values
PDI	23.26%	24.77%	0.06
hPDI	19.14%	28.03%	<0.001*
uPDI	27.03%	21.29%	<0.001*
DASH	20.57%	29.21%	<0.001*
MedDiet	21.38%	27.41%	<0.001*
PHDI	18.92%	29.62%	<0.001*
LCD	25.25%	22.01%	<0.001*
HLCD	20.27%	28.38%	<0.001*
ULCD	28.81%	20.28%	<0.001*
EDIP	26.33%	21.48%	<0.001*
EDIH	28.39%	19.85%	<0.001*
UPF	24.65%	23.12%	0.04

Data were presented as percentage (%) for categorical variables. P values were calculated using the chi-square test. * Indicated Bonferroni-adjusted P < 0.05.

DASH, Dietary Approaches to Stop Hypertension; EDIH, empirical dietary index for hyperinsulinemia; EDIP, empirical dietary inflammation pattern; HLCD, healthy low-carbohydrate diet; hPDI, healthy plant-based diet index; LCD, low-carbohydrate diet; MedDiet, Mediterranean diet; MET, metabolic equivalent; PDI, plant-based diet index; PHDI, Planetary Health Diet Index; ULCD, unhealthy low-carbohydrate diet; uPDI, unhealthy plant-based diet index; UPF, ultra-processed food

© 2026 Xia T et al. JAMA Network Open.

eTable 8. Associations [HR (95% CI)] of dietary patterns (Quintile 5 vs. 1) with obesity risk during menopause in the Nurse Health Study II

	Quintile 1	Quintile 5 vs. 1<br>Quintile 5	P value
PDI			
Cases/ Person-years	1,261/66,049	861/65,216	
Age and race model	Ref	0.69 (0.63, 0.75)	<0.001*
Multivariable model	Ref	0.74 (0.67, 0.81)	<0.001*
Multivariable + BMI model	Ref	0.98 (0.90, 1.08)	0.76
hPDI			
Cases/ Person-years	1,243/67,793	889/67,092	
Age and race model	Ref	0.73 (0.67, 0.79)	<0.001*
Multivariable model	Ref	0.92 (0.84, 1.00)	0.06
Multivariable + BMI model	Ref	1.03 (0.94, 1.13)	0.52
uPDI			
Cases/ Person-years	1,029/68,521	1,216/63,610	
Age and race model	Ref	1.26 (1.16, 1.37)	<0.001*
Multivariable model	Ref	0.89 (0.81, 0.97)	0.01
Multivariable + BMI model	Ref	1.05 (0.96, 1.15)	0.26
DASH			
Cases/ Person-years	1,277/60,928	787/70,981	
Age and race model	Ref	0.53 (0.48, 0.58)	<0.001*
Multivariable model	Ref	0.71 (0.65, 0.78)	<0.001*
Multivariable + BMI model	Ref	0.85 (0.77, 0.93)	0.001*
MedDiet			
Cases/ Person-years	1,549/69,609	799/66,059	
Age and race model	Ref	0.54 (0.50, 0.59)	<0.001*
Multivariable model	Ref	0.72 (0.66, 0.79)	<0.001*
Multivariable + BMI model	Ref	0.82 (0.75, 0.90)	<0.001*
PHDI			
Cases/ Person-years	1,511/59,446	594/73,778	
Age and race model	Ref	0.31 (0.28, 0.34)	<0.001*
Multivariable model	Ref	0.39 (0.36, 0.43)	<0.001*

© 2026 Xia T et al. JAMA Network Open.

Multivariable + BMI model	Ref	0.46 (0.42, 0.51)	<0.001*
LCD			
Cases/ Person-years	929/68,153	1,411/61,092	
Age and race model	Ref	1.71 (1.57, 1.85)	<0.001*
Multivariable model	Ref	1.84 (1.69, 2.00)	<0.001*
Multivariable + BMI model	Ref	1.32 (1.21, 1.43)	<0.001*
HLCD			
Cases/ Person-years	1,109/63,036	885/69,569	
Age and race model	Ref	0.73 (0.67, 0.80)	<0.001*
Multivariable model	Ref	0.98 (0.90, 1.08)	0.71
Multivariable + BMI model	Ref	0.99 (0.91, 1.08)	0.84
ULCD			
Cases/ Person-years	716/68,874	1,515/61,725	
Age and race model	Ref	2.38 (2.18, 2.60)	<0.001*
Multivariable model	Ref	1.98 (1.80, 2.16)	<0.001*
Multivariable + BMI model	Ref	1.40 (1.28, 1.53)	<0.001*
EDIP			
Cases/ Person-years	792/69862	1,538/61,835	
Age and race model	Ref	2.22 (2.04, 2.42)	<0.001*
Multivariable model	Ref	2.04 (1.87, 2.23)	<0.001*
Multivariable + BMI model	Ref	1.55 (1.42, 1.70)	<0.001*
EDIH			
Cases/ Person-years	622/71,377	1,614/61,734	
Age and race model	Ref	3.01 (2.74, 3.30)	<0.001*
Multivariable model	Ref	2.97 (2.69, 3.27)	<0.001*
Multivariable + BMI model	Ref	1.96 (1.77, 2.16)	<0.001*
UPF			
Cases/ Person-years	911/67,143	1,432/64,601	
Age and race model	Ref	1.63 (1.50, 1.78)	<0.001*
Multivariable model	Ref	1.74 (1.59, 1.90)	<0.001*
Multivariable + BMI model	Ref	1.34 (1.23, 1.46)	<0.001*

Cox proportional hazards regression models were used. * indicated Bonferroni-adjusted P < 0.05.

© 2026 Xia T et al. JAMA Network Open.

Age and race model: adjust for baseline age (≤45, >45 years), race (White; Asian, American Indian/Alaska Native, Native Hawaiian/Other; Black; Hispanic). Multivariable model: adjust for baseline age (≤45, >45 years), race (White; Asian, American Indian/Alaska Native, Native Hawaiian/Other; Black; Hispanic), marital status (never, ever), income (<50k, 50k–99k, ≥100k, missing), postmenopausal hormone use (never, ever, missing), parity (nulliparous, 1 and 2, or ≥3 births), smoking status (never, ever), alcohol (g/day; excluding EDIP, EDIH, and MedDiet scores, where alcohol is included in the scoring criteria), total energy intake (<median, ≥median), and physical activity (<1000, ≥1000 MET-mins/week). Multivariable and BMI model: adjust for baseline age (≤45, >45 years), race (White; Asian, American Indian/Alaska Native, Native Hawaiian/Other; Black; Hispanic), marital status (never, ever), income (<50k, 50k–99k, ≥100k, missing), postmenopausal hormone use (never, ever, missing), parity (nulliparous, 1 and 2, or ≥3 births), smoking status (never, ever), alcohol (g/day;), total energy intake (<median, ≥median), physical activity (<1000, ≥1000 MET-mins/week), and baseline body mass index (>18 and <25 kg/m2, ≥25 and <30 kg/m2).

BMI: body mass index; DASH, Dietary Approaches to Stop Hypertension; EDIH, empirical dietary index for hyperinsulinemia; EDIP, empirical dietary inflammation pattern; HLCD, healthy low-carbohydrate diet; hPDI, healthy plant-based diet index; LCD, low-carbohydrate diet; MedDiet, Mediterranean diet; MET, metabolic equivalent; PDI, plant-based diet index; PHDI, Planetary Health Diet Index; ULCD, unhealthy low-carbohydrate diet; uPDI, unhealthy plant-based diet index; UPF, ultra-processed food

© 2026 Xia T et al. JAMA Network Open.

eTable 9. Associations [HR (95% CI)] of dietary patterns (per SD) with obesity risk during menopause in the Nurse Health Study II

		**Per SD**<br>(Cases/ Person-years:<br>5,214/ 340,122)		
PDI				
	Age and race model	0.86	(0.84,0.89)	<0.001*
	Multivariable model	0.87	(0.85,0.90)	<0.001*
	Multivariable + BMI model	0.97	(0.94,1.00)	0.04
hPDI				
	Age and race model	0.9	(0.87,0.92)	<0.001*
	Multivariable model	0.98	(0.95,1.01)	0.23
	Multivariable + BMI model	1.03	(1.00,1.06)	0.06
uPDI				
	Age and race model	1.08	(1.05,1.11)	<0.001*
	Multivariable model	0.95	(0.92,0.98)	<0.001*
	Multivariable + BMI model	1.01	(0.98,1.04)	0.72
DASH				
	Age and race model	0.79	(0.76,0.81)	<0.001*
	Multivariable model	0.87	(0.84,0.89)	<0.001*
	Multivariable + BMI model	0.92	(0.89,0.95)	<0.001*
MedDiet				
	Age and race model	0.81	(0.79,0.83)	<0.001*
	Multivariable model	0.91	(0.88,0.93)	<0.001*
	Multivariable + BMI model	0.95	(0.92,0.98)	<0.001*
PHDI				
	Age and race model	0.65	(0.63,0.67)	<0.001*
	Multivariable model	0.71	(0.68,0.73)	<0.001*
	Multivariable + BMI model	0.74	(0.72,0.76)	<0.001*
LCD				
	Age and race model	1.28	(1.25,1.32)	<0.001*
	Multivariable model	1.3	(1.27,1.34)	<0.001*

© 2026 Xia T et al. JAMA Network Open.

	Multivariable + BMI model	1.16	(1.13,1.19)	<0.001*
HLCD				
	Age and race model	0.89	(0.87,0.92)	<0.001*
	Multivariable model	0.99	(0.97,1.02)	0.63
	Multivariable + BMI model	0.99	(0.97,1.02)	0.70
ULCD				
	Age and race model	1.37	(1.33,1.41)	<0.001*
	Multivariable model	1.27	(1.24,1.31)	<0.001*
	Multivariable + BMI model	1.15	(1.11,1.18)	<0.001*
EDIP				
	Age and race model	1.35	(1.32,1.39)	<0.001*
	Multivariable model	1.3	(1.26,1.33)	<0.001*
	Multivariable + BMI model	1.17	(1.14,1.20)	<0.001*
EDIH				
	Age and race model	1.45	(1.42,1.49)	<0.001*
	Multivariable model	1.45	(1.41,1.49)	<0.001*
	Multivariable + BMI model	1.26	(1.23,1.30)	<0.001*
UPF				
	Age and race model	1.22	(1.19, 1.25)	<0.001*
	Multivariable model	1.23	(1.20,1.27)	<0.001*
	Multivariable + BMI model	1.13	(1.09,1.16)	<0.001*

Cox proportional hazards regression models were used. * indicated Bonferroni-adjusted P < 0.05.
Age and race model: adjust for baseline age (≤45, >45 years), race (White; Asian, American Indian/Alaska Native, Native Hawaiian/Other; Black; Hispanic). Multivariable model: adjust for baseline age (≤45, >45 years), race (White; Asian, American Indian/Alaska Native, Native Hawaiian/Other; Black; Hispanic), marital status (never, ever), income (<50k, 50k–99k, ≥100k, missing), postmenopausal hormone use (never, ever, missing), parity (nulliparous, 1 and 2, or ≥3 births), smoking status (never, ever), alcohol (g/day; excluding EDIP, EDIH, and MedDiet scores, where alcohol is included in the scoring criteria), total energy intake (<median, ≥median), and physical activity (<1000, ≥1000 MET-mins/week). Multivariable and BMI model: adjust for baseline age (≤45, >45 years), race (White; Asian, American Indian/Alaska Native, Native Hawaiian/Other; Black; Hispanic), marital status (never, ever), income (<50k, 50k–99k, ≥100k, missing), postmenopausal hormone use (never, ever, missing), parity (nulliparous, 1 and 2, or ≥3 births), smoking status (never, ever), alcohol (g/day;), total energy intake (<median, ≥median), physical activity (<1000, ≥1000 MET-mins/week), and baseline body mass index (>18 and <25 kg/m2, ≥25 and <30 kg/m2).
BMI: body mass index; DASH, Dietary Approaches to Stop Hypertension; EDIH, empirical dietary index for hyperinsulinemia; EDIP, empirical dietary inflammation pattern; HLCD, healthy low-carbohydrate diet; hPDI, healthy plant-based diet index; LCD, low-carbohydrate diet; MedDiet, Mediterranean diet; MET, metabolic equivalent; PDI, plant-based diet index; PHDI, Planetary Health Diet Index; ULCD, unhealthy low-carbohydrate diet; uPDI, unhealthy plant-based diet index; UPF, ultra-processed food

© 2026 Xia T et al. JAMA Network Open.

eTable 10. Associations of dietary patterns (quintile 5 vs. 1) with weight change during menopause in the Nurse Health Study II, diet assessed by average of two dietary scores within each weight change interval

	**Estimate**	**95%CI**		***P***
		**Lower bound**	**Upper bound**	
PDI	-0.19	-0.21	-0.17	<0.001*
hPDI	-0.003	-0.02	0.02	0.81
uPDI	-0.07	-0.09	-0.05	<0.001*
DASH	-0.11	-0.13	-0.09	<0.001*
MedDiet	-0.08	-0.10	-0.06	<0.001*
PHDI	-0.11	-0.13	-0.08	<0.001*
LCD	0.16	0.14	0.18	<0.001*
HLCD	-0.01	-0.03	0.02	0.59
ULCD	0.15	0.13	0.17	<0.001*
EDIP	0.15	0.13	0.17	<0.001*
EDIH	0.21	0.19	0.24	<0.001*
UPF	0.09	0.07	0.11	<0.001*

Adjusted mean annualized weight change (kg/year) and 95%CI were reported, which were estimated using generalized estimating equations with repeated measures to account for within-person correlation across follow-up intervals, specifying an unstructured working correlation matrix. * indicated Bonferroni-adjusted P < 0.05.

Diet assessed by average of two dietary scores within each weight change interval.

Model adjust for baseline age (≤45, >45 years), race (White; Asian, American Indian/Alaska Native, Native Hawaiian/Other; Black; Hispanic), marital status (never, ever), income (<50k, 50k–99k, ≥100k, missing), postmenopausal hormone use (never, ever, missing), parity (nulliparous, 1 and 2, or ≥3 births), smoking status (never, ever), alcohol (g/day), total energy intake (<median, ≥median), physical activity (<1000, ≥1000 MET-mins/week), and each interval baseline body mass index (>18 and <25 kg/m^2, ≥25 kg/m^2). For MedDiet, EDIP, and EDIH, the alcohol was not adjusted for. DASH, Dietary Approaches to Stop Hypertension; EDIH, empirical dietary index for hyperinsulinemia; EDIP, empirical dietary inflammation pattern; HLCD, healthy low-carbohydrate diet; hPDI, healthy plant-based diet index; LCD, low-carbohydrate diet; MedDiet, Mediterranean diet; MET, metabolic equivalent; PDI, plant-based diet index; PHDI, Planetary Health Diet Index; ULCD, unhealthy low-carbohydrate diet; uPDI, unhealthy plant-based diet index; UPF, ultra-processed food

© 2026 Xia T et al. JAMA Network Open.

eTable 11. Associations of dietary patterns (quintile 5 vs. 1) with weight gain and obesity risk during menopause in the Nurse Health Study II, physical activity tertiles adjusted for

	Weight gain				Obesity			
	Estimate	95%CI		*P*	HR	95%CI		*P*
		Lower bound	Upper bound			Lower bound	Upper bound	
PDI	-0.23	-0.25	-0.21	<0.001	0.99	0.90	1.09	0.90
hPDI	-0.06	-0.09	-0.04	<0.001	1.07	0.97	1.17	0.17
uPDI	-0.04	-0.06	-0.01	0.002	1.02	0.93	1.11	0.74
DASH	-0.18	-0.20	-0.16	<0.001	0.88	0.80	0.97	0.01
MedDiet	-0.13	-0.15	-0.11	<0.001	0.86	0.78	0.94	0.001
PHDI	-0.15	-0.18	-0.13	<0.001	0.48	0.43	0.53	<0.001
LCD	0.18	0.16	0.20	<0.001	1.32	1.21	1.43	<0.001
HLCD	-0.04	-0.07	-0.02	<0.001	1.02	0.94	1.12	0.61
ULCD	0.20	0.18	0.22	<0.001	1.36	1.24	1.49	<0.001
EDIP	0.19	0.17	0.22	<0.001	1.52	1.40	1.66	<0.001
EDIH	0.27	0.25	0.30	<0.001	1.91	1.73	2.10	<0.001
UPF	0.10	0.08	0.12	<0.001	1.33	1.22	1.45	<0.001

For diet and weight change: Adjusted mean annualized weight change (kg/year) and 95%CI were reported, which were estimated using generalized estimating equations with repeated measures to account for within-person correlation across follow-up intervals, specifying an unstructured working correlation matrix. * indicated Bonferroni-adjusted P < 0.05. Model adjust for baseline age (≤45, >45 years), race (White; Asian, American Indian/Alaska Native, Native Hawaiian/Other; Black; Hispanic), marital status (never, ever), income (<50k, 50k–99k, ≥100k, missing), postmenopausal hormone use (never, ever, missing), parity (nulliparous, 1 and 2, or ≥3 births), smoking status (never, ever), alcohol (g/day), total energy intake (<median, ≥median), physical activity (tertiles), and each interval baseline body mass index (>18 and <25 kg/m^2, ≥25 kg/m^2). For MedDiet, EDIP, and EDIH, the alcohol was not adjusted for.
For diet and obesity risk: Associations between dietary patterns (quintile 5 vs. 1) and obesity were analyzed using Cox proportional harazrds regression models. Hazard ratio (95% CI), and P-value were reported. * indicated Bonferroni-adjusted P < 0.05. Model adjust for baseline age (≤45, >45 years), race (White; Asian, American Indian/Alaska Native, Native Hawaiian/Other; Black; Hispanic), marital status (never, ever), income (<50k, 50k–99k, ≥100k, missing), postmenopausal hormone use (never, ever, missing), parity (nulliparous, 1 and 2, or ≥3 births), smoking status (never, ever), alcohol (g/day), total energy intake (<median, ≥median), physical activity (tertiles), and baseline body mass index (>18 and <25 kg/m^2, ≥25 and <30 kg/m^2). For MedDiet, EDIP, and EDIH, the alcohol was not adjusted for. DASH, Dietary Approaches to Stop Hypertension; EDIH, empirical dietary index for hyperinsulinemia; EDIP, empirical dietary inflammation pattern; HLCD, healthy low-carbohydrate diet; hPDI, healthy plant-based diet index; LCD, low-carbohydrate diet; MedDiet, Mediterranean diet; MET, metabolic equivalent; PDI, plant-based diet index; PHDI, Planetary Health Diet Index; ULCD, unhealthy low-carbohydrate diet; uPDI, unhealthy plant-based diet index; UPF, ultra-processed food

© 2026 Xia T et al. JAMA Network Open.

eTable 12. Subgroup analyses of associations of dietary patterns (quintile 5 vs. 1) with weight gain during menopause in the Nurse Health Study II

Diet Pattern	Subgroup	β	Lower 95% CI	Upper 95% CI	P for interaction	Subgroup	β	Lower 95% CI	Upper 95% CI	P for interaction
PDI	age≤45 years	-0.24	-0.27	-0.21	0.21	18<BMI<25 kg/m^2	-0.25	-0.27	-0.22	0.60
PDI	age>45 years	-0.23	-0.26	-0.20		≥25 kg/m^2	-0.23	-0.27	-0.19	
hPDI	age≤45 years	-0.06	-0.09	-0.03	0.93	18<BMI<25 kg/m^2	-0.11	-0.14	-0.08	0.12
hPDI	age>45 years	-0.09	-0.12	-0.06		≥25 kg/m^2	-0.04	-0.08	-0.001	
uPDI	age≤45 years	-0.03	-0.06	0.002	0.75	18<BMI<25 kg/m^2	-0.02	-0.05	0.01	0.75
uPDI	age>45 years	-0.02	-0.05	0.01		≥25 kg/m^2	-0.03	-0.07	0.01	
DASH	age≤45 years	-0.16	-0.20	-0.13	0.09	18<BMI<25 kg/m^2	-0.22	-0.25	-0.19	0.001
DASH	age>45 years	-0.22	-0.25	-0.19		≥25 kg/m^2	-0.16	-0.20	-0.12	
MedDiet	age≤45 years	-0.14	-0.17	-0.10	0.80	18<BMI<25 kg/m^2	-0.16	-0.18	-0.13	0.03
MedDiet	age>45 years	-0.16	-0.19	-0.13		≥25 kg/m^2	-0.13	-0.17	-0.10	
PHDI	age≤45 years	-0.18	-0.21	-0.15	0.21	18<BMI<25 kg/m^2	-0.19	-0.21	-0.16	0.57
PHDI	age>45 years	-0.16	-0.19	-0.13		≥25 kg/m^2	-0.15	-0.19	-0.11	
LCD	age≤45 years	0.20	0.17	0.23	0.52	18<BMI<25 kg/m^2	0.19	0.17	0.22	0.07
LCD	age>45 years	0.17	0.14	0.20		≥25 kg/m^2	0.18	0.14	0.22	
HLCD	age≤45 years	-0.05	-0.08	-0.02	0.66	18<BMI<25 kg/m^2	-0.09	-0.11	-0.06	0.004
HLCD	age>45 years	-0.06	-0.09	-0.04		≥25 kg/m^2	-0.02	-0.06	0.02	
ULCD	age≤45 years	0.20	0.17	0.23	0.52	18<BMI<25 kg/m^2	0.24	0.21	0.27	0.006
ULCD	age>45 years	0.21	0.18	0.24		≥25 kg/m^2	0.16	0.13	0.20	
EDIP	age≤45 years	0.20	0.17	0.23	0.80	18<BMI<25 kg/m^2	0.22	0.19	0.25	0.70
EDIP	age>45 years	0.20	0.17	0.23		≥25 kg/m^2	0.19	0.15	0.22	
EDIH	age≤45 years	0.29	0.26	0.32	0.42	18<BMI<25 kg/m^2	0.31	0.28	0.34	0.30
EDIH	age>45 years	0.27	0.24	0.31		≥25 kg/m^2	0.26	0.22	0.30	
UPF	age≤45 years	0.12	0.09	0.16	0.15	18<BMI<25 kg/m^2	0.10	0.07	0.13	0.12

© 2026 Xia T et al. JAMA Network Open.

UPF	age>45 years	0.09	0.06	0.12		≥25 kg/m^2	0.12	0.08	0.15	
Diet Pattern	Subgroup	Estimate	Lower CI	UpperCI	P for interaction	Subgroup	Estimate	LowerCI	UpperCI	P for interaction
PDI	PA<1000 MET-mins/week	-0.24	-0.28	-0.21	0.26	never smoking	-0.23	-0.25	-0.20	0.27
PDI	PA≥1000 MET-mins/week	-0.22	-0.25	-0.19		ever smoking	-0.25	-0.29	-0.21	
hPDI	PA<1000 MET-mins/week	-0.05	-0.08	-0.01	0.02	never smoking	-0.08	-0.10	-0.05	0.07
hPDI	PA≥1000 MET-mins/week	-0.10	-0.13	-0.07		ever smoking	-0.07	-0.11	-0.03	
uPDI	PA<1000 MET-mins/week	-0.07	-0.11	-0.04	0.001	never smoking	-0.01	-0.04	0.02	0.34
uPDI	PA≥1000 MET-mins/week	0.02	-0.02	0.05		ever smoking	-0.05	-0.09	0.01	
DASH	PA<1000 MET-mins/week	-0.17	-0.20	-0.13	0.33	never smoking	-0.19	-0.22	-0.17	0.94
DASH	PA≥1000 MET-mins/week	-0.20	-0.23	-0.17		ever smoking	-0.19	-0.23	-0.16	
MedDiet	PA<1000 MET-mins/week	-0.13	-0.17	-0.10	0.12	never smoking	-0.14	-0.17	-0.12	0.77
MedDiet	PA≥1000 MET-mins/week	-0.15	-0.18	-0.12		ever smoking	-0.15	-0.19	-0.12	
PHDI	PA<1000 MET-mins/week	-0.15	-0.18	-0.11	0.52	never smoking	-0.15	-0.18	-0.13	0.19
PHDI	PA≥1000 MET-mins/week	-0.19	-0.21	-0.16		ever smoking	-0.19	-0.23	-0.16	
LCD	PA<1000 MET-mins/week	0.19	0.16	0.22	0.59	never smoking	0.18	0.16	0.21	0.64
LCD	PA≥1000 MET-mins/week	0.17	0.15	0.20		ever smoking	0.18	0.15	0.22	
HLCD	PA<1000 MET-mins/week	-0.04	-0.07	-0.004	0.01	never smoking	-0.05	-0.08	-0.03	0.55
HLCD	PA≥1000 MET-mins/week	-0.07	-0.10	-0.04		ever smoking	-0.06	-0.10	-0.02	
ULCD	PA<1000 MET-mins/week	0.20	0.16	0.23	0.92	never smoking	0.20	0.17	0.23	0.64
ULCD	PA≥1000 MET-mins/week	0.21	0.18	0.24		ever smoking	0.22	0.18	0.25	
EDIP	PA<1000 MET-mins/week	0.19	0.16	0.23	0.02	never smoking	0.18	0.16	0.21	0.16
EDIP	PA≥1000 MET-mins/week	0.21	0.18	0.24		ever smoking	0.23	0.19	0.27	

© 2026 Xia T et al. JAMA Network Open.

EDIH	PA<1000 MET-mins/week	0.28	0.24	0.31	0.02	never smoking	0.28	0.26	0.31	0.62
EDIH	PA≥1000 MET-mins/week	0.28	0.25	0.31		ever smoking	0.27	0.23	0.31	
UPF	PA<1000 MET-mins/week	0.12	0.09	0.16	0.03	never smoking	0.10	0.07	0.13	0.27
UPF	PA≥1000 MET-mins/week	0.09	0.06	0.12		ever smoking	0.12	0.08	0.15	

Diet Pattern	Subgroup	Estimate	Lower CI	UpperCI	P for interaction	Subgroup	Estimate	LowerCI	UpperCI	P for interaction
PDI	Alcohol<3g/day	-0.24	-0.27	-0.21	0.44	never used PMH	-0.25	-0.29	-0.21	0.17
PDI	Alcohol≥3g/day	-0.22	-0.25	-0.19		used PMH	-0.23	-0.28	-0.17	
hPDI	Alcohol<3g/day	-0.07	-0.10	-0.04	0.54	never used PMH	-0.09	-0.13	-0.05	0.30
hPDI	Alcohol≥3g/day	-0.08	-0.11	-0.05		used PMH	-0.06	-0.11	-0.001	
uPDI	Alcohol<3g/day	-0.02	-0.05	0.01	0.37	never used PMH	-0.04	-0.08	0.01	0.68
uPDI	Alcohol≥3g/day	-0.04	-0.07	-0.004		used PMH	-0.03	-0.09	0.02	
DASH	Alcohol<3g/day	-0.18	-0.21	-0.15	0.06	never used PMH	-0.19	-0.23	-0.15	0.61
DASH	Alcohol≥3g/day	-0.20	-0.23	-0.17		used PMH	-0.18	-0.24	-0.13	
MedDiet	Alcohol<3g/day	-0.12	-0.16	-0.09	0.82	never used PMH	-0.14	-0.18	-0.10	0.48
MedDiet	Alcohol≥3g/day	-0.13	-0.16	-0.10		used PMH	-0.14	-0.19	-0.09	
PHDI	Alcohol<3g/day	-0.17	-0.20	-0.14	0.005	never used PMH	-0.15	-0.19	-0.11	0.08
PHDI	Alcohol≥3g/day	-0.16	-0.19	-0.13		used PMH	-0.17	-0.22	-0.11	
LCD	Alcohol<3g/day	0.19	0.16	0.22	0.87	never used PMH	0.19	0.15	0.23	0.72
LCD	Alcohol≥3g/day	0.19	0.16	0.21		used PMH	0.20	0.15	0.25	
HLCD	Alcohol<3g/day	-0.05	-0.08	-0.02	0.76	never used PMH	-0.05	-0.08	-0.01	0.02
HLCD	Alcohol≥3g/day	-0.05	-0.08	-0.02		used PMH	-0.02	-0.08	0.03	
ULCD	Alcohol<3g/day	0.21	0.18	0.24	0.18	never used PMH	0.20	0.16	0.24	0.28
ULCD	Alcohol≥3g/day	0.21	0.18	0.24		used PMH	0.21	0.16	0.26	
EDIP	Alcohol<3g/day	0.16	0.12	0.20	0.01	never used PMH	0.17	0.13	0.21	0.0003*
EDIP	Alcohol≥3g/day	0.21	0.18	0.25		used PMH	0.24	0.19	0.29	

© 2026 Xia T et al. JAMA Network Open.

EDIH	Alcohol<3g/day	0.28	0.24	0.31	0.62	never used PMH	0.24	0.19	0.28	0.04
EDIH	Alcohol≥3g/day	0.26	0.23	0.29		used PMH	0.30	0.24	0.35	
UPF	Alcohol<3g/day	0.11	0.08	0.14	0.14	never used PMH	0.12	0.08	0.16	0.39
UPF	Alcohol≥3g/day	0.09	0.06	0.13		used PMH	0.04	-0.01	0.10	

Associations between dietary patterns (quintile 5 vs. 1) and weight gain in subgroups. * indicated Bonferroni-adjusted P < 0.05 for the interaction. Models were adjusted for baseline age (≤45, >45 years), race (White; Asian, American Indian/Alaska Native, Native Hawaiian/Other; Black; Hispanic), marital status (never, ever), income (<50k, 50k–99k, ≥100k, missing), postmenopausal hormone use (never, ever, missing), parity (nulliparous, 1 and 2, or ≥3 births), smoking status (never, ever), alcohol (g/day), total energy intake (<median, ≥median), physical activity (<1000, ≥1000 MET-mins/week), and each interval baseline body mass index (>18 and <25 kg/m^2, ≥25 kg/m^2). For MedDiet, EDIP, EDIH, the alcohol was not adjusted for. When the association was stratified by one variable, that variable was not adjusted for in the model. Effect modification was tested using Wald χ^2. BMI, body mass index; CI, confidence interval; DASH, Dietary Approaches to Stop Hypertension; EDIH, empirical dietary index for hyperinsulinemia; EDIP, empirical dietary inflammation pattern; HLCD, healthy low-carbohydrate diet; hPDI, healthy plant-based diet index; LCD, low-carbohydrate diet; MedDiet, Mediterranean diet; MET, metabolic equivalent; PDI, plant-based diet index; PHDI, Planetary Health Diet Index; ULCD, unhealthy low-carbohydrate diet; uPDI, unhealthy plant-based diet index; UPF, ultra-processed food

© 2026 Xia T et al. JAMA Network Open.

eTable 13. Correlations of dietary pattern scores with protein intake

Diet Pattern	Total protein	Vegetable protein	Animal protein
PDI	-0.38	0.36	-0.50
hPDI	0.10	0.51	-0.11
uPDI	-0.23	-0.32	-0.09
DASH	-0.02	0.50	-0.20
MedDiet	-0.01	0.51	-0.20
PHDI	-0.07	0.66	-0.31
LCD	0.49	-0.22	0.54
HLCD	0.11	0.61	-0.13
ULCD	0.34	-0.62	0.57
EDIP	0.10	-0.20	0.17
EDIH	0.16	-0.32	0.27
UPF	-0.09	-0.07	-0.06

PDI, plant-based diet index; hPDI, healthy plant-based diet index; uPDI, unhealthy plant-based diet index; DASH, Dietary Approaches to Stop Hypertension; MedDiet, Mediterranean diet; PHDI, Planetary Health Diet Index; LCD, low-carbohydrate diet; HLCD, healthy low-carbohydrate diet; ULCD, unhealthy low-carbohydrate diet; EDIP, empirical dietary inflammatory pattern; EDIH, empirical dietary index for hyperinsulinemia; UPF, ultra-processed food.

© 2026 Xia T et al. JAMA Network Open.

eTable 14. Mean protein intake (grams/day) in quintile 1 versus quintile 5 of each dietary pattern

Diet Pattern	Protein	Mean of protein in Quintile 1 of diet pattern	Mean of protein in Quintile 5 of diet pattern	P value
PDI	Total protein	19.65	16.37	<0.001*
PDI	Animal protein	14.44	9.76	<0.001*
PDI	Vegetable protein	5.21	6.61	<0.001*
hPDI	Total protein	17.38	18.10	<0.001*
hPDI	Animal protein	12.34	11.05	<0.001*
hPDI	Vegetable protein	5.04	7.05	<0.001*
uPDI	Total protein	18.82	16.80	<0.001*
uPDI	Animal protein	12.43	11.54	<0.001*
uPDI	Vegetable protein	6.38	5.26	<0.001*
DASH	Total protein	17.84	17.63	<0.001*
DASH	Animal protein	12.85	10.71	<0.001*
DASH	Vegetable protein	4.99	6.92	<0.001*
MedDiet	Total protein	17.85	17.67	<0.001*
MedDiet	Animal protein	12.85	10.84	<0.001*
MedDiet	Vegetable protein	5.00	6.83	<0.001*
PHDI	Total protein	17.97	17.40	<0.001*
PHDI	Animal protein	13.23	10.14	<0.001*
PHDI	Vegetable protein	4.75	7.26	<0.001*
LCD	Total protein	15.82	20.18	<0.001*
LCD	Animal protein	9.51	14.74	<0.001*
LCD	Vegetable protein	6.31	5.44	<0.001*
HLCD	Total protein	17.09	18.04	<0.001*
HLCD	Animal protein	12.30	10.91	<0.001*
HLCD	Vegetable protein	4.80	7.12	<0.001*
ULCD	Total protein	16.47	19.47	<0.001*
ULCD	Animal protein	9.23	14.63	<0.001*
ULCD	Vegetable protein	7.24	4.85	<0.001*
EDIP	Total protein	17.34	18.07	<0.001*
EDIP	Animal protein	11.21	12.67	<0.001*
EDIP	Vegetable protein	6.13	5.39	<0.001*
EDIH	Total protein	16.90	18.21	<0.001*
EDIH	Animal protein	10.38	12.98	<0.001*
EDIH	Vegetable protein	6.52	5.23	<0.001*
UPF	Total protein	18.22	17.47	<0.001*

© 2026 Xia T et al. JAMA Network Open.

UPF	Animal protein	12.17	11.75	<0.001*
UPF	Vegetable protein	6.05	5.73	<0.001*

Values are means (grams/day). P values compare mean of protein in quintile 1 and quintile 5 of each dietary pattern. * indicated Bonferroni-adjusted P < 0.05. PDI, plant-based diet index; hPDI, healthy plant-based diet index; uPDI, unhealthy plant-based diet index; DASH, Dietary Approaches to Stop Hypertension; MedDiet, Mediterranean diet; PHDI, Planetary Health Diet Index; LCD, low-carbohydrate diet; HLCD, healthy low-carbohydrate diet; ULCD, unhealthy low-carbohydrate diet; EDIP, empirical dietary inflammatory pattern; EDIH, empirical dietary index for hyperinsulinemia; UPF, ultra-processed food

© 2026 Xia T et al. JAMA Network Open.

eTable 15. Association of dietary patterns (quintile 5 vs. 1) with weight gain after adjustment for total protein intake

Diet pattern	Estimate	95% CI	P value
PDI	-0.16	(-0.18, -0.13)	<0.001*
hPDI	-0.09	(-0.11, -0.07)	<0.001*
uPDI	0.07	(0.05, 0.10)	<0.001*
DASH	-0.20	(-0.22, -0.18)	<0.001*
MedDiet	-0.16	(-0.18, -0.14)	<0.001*
PHDI	-0.15	(-0.17, -0.13)	<0.001*
LCD	0.08	(0.05, 0.10)	<0.001*
HLCD	-0.09	(-0.11, -0.07)	<0.001*
ULCD	0.13	(0.11, 0.16)	<0.001*
EDIP	0.18	(0.16, 0.20)	<0.001*
EDIH	0.24	(0.21, 0.26)	<0.001*
UPF	0.12	(0.10, 0.15)	<0.001*

Adjusted mean annualized weight change (kg/year) and 95%CI were reported, which were estimated using generalized estimating equations with repeated measures to account for within-person correlation across follow-up intervals, specifying an unstructured working correlation matrix. Models were adjusted for baseline age (≤45, >45 years), race (White; Asian, American Indian/Alaska Native, Native Hawaiian/Other; Black; Hispanic), marital status (never, ever), income (<50k, 50k–99k, ≥100k, missing), postmenopausal hormone use (never, ever, missing), parity (nulliparous, 1 and 2, or ≥3 births), smoking status (never, ever), alcohol (g/day), total protein intake (grams/day), total energy intake (<median, ≥median), physical activity (<1000, ≥1000 MET-mins/week), and each interval baseline body mass index (>18 and <25 kg/m2, ≥25 kg/m2). For MedDiet, EDIP, EDIH, the alcohol was not adjusted for. * indicated Bonferroni-adjusted P < 0.05. PDI, plant-based diet index; hPDI, healthy plant-based diet index; uPDI, unhealthy plant-based diet index; DASH, Dietary Approaches to Stop Hypertension; MedDiet, Mediterranean diet; PHDI, Planetary Health Diet Index; LCD, low-carbohydrate diet; HLCD, healthy low-carbohydrate diet; ULCD, unhealthy low-carbohydrate diet; EDIP, empirical dietary inflammatory pattern; EDIH, empirical dietary index for hyperinsulinemia; UPF, ultra-processed food.

© 2026 Xia T et al. JAMA Network Open.

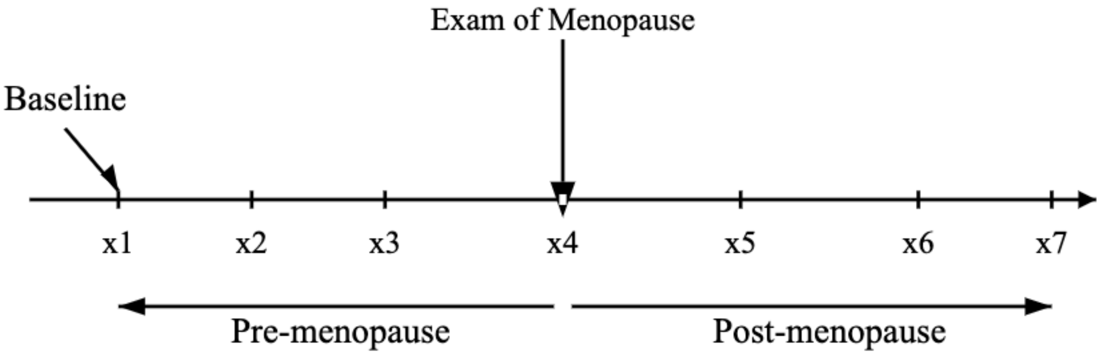

eFigure 1. Study timeline for weight change analysis around menopause. The menopause questionnaire (e.g., questionnaire x4) is positioned at the midpoint of the analysis. We included data from three questionnaires before and three questionnaires after menopause, each spaced two years apart. For example, questionnaire x4 is roughly six years after the baseline questionnaire (questionnaire x1).

© 2026 Xia T et al. JAMA Network Open.

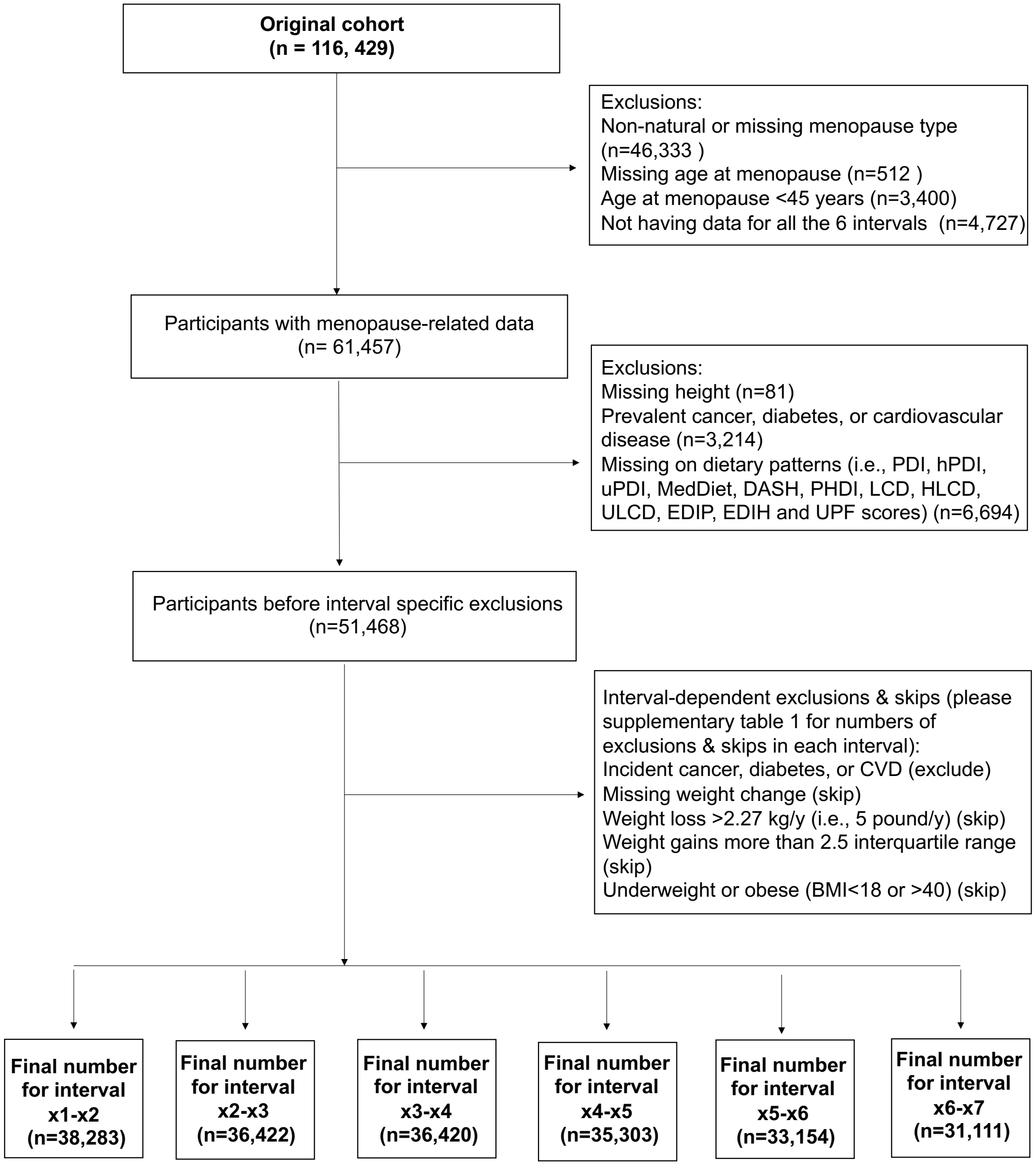

eFigure 2. Sample selection of weight change analysis. BMI, body mass index; CVD, cardiovascular disease; DASH, Dietary Approaches to Stop Hypertension; EDIH, empirical dietary index for hyperinsulinemia; EDIP, empirical dietary inflammation pattern; HLCD, healthy low-carbohydrate diet; hPDI, healthy plant-based diet index; LCD, low-carbohydrate diet; MedDiet, Mediterranean diet; MET, metabolic equivalent; PDI, plant-based diet index; PHDI, Planetary Health Diet Index; ULCD, unhealthy low-carbohydrate diet; uPDI, unhealthy plant-based diet index; UPF, ultra-processed food

© 2026 Xia T et al. JAMA Network Open.

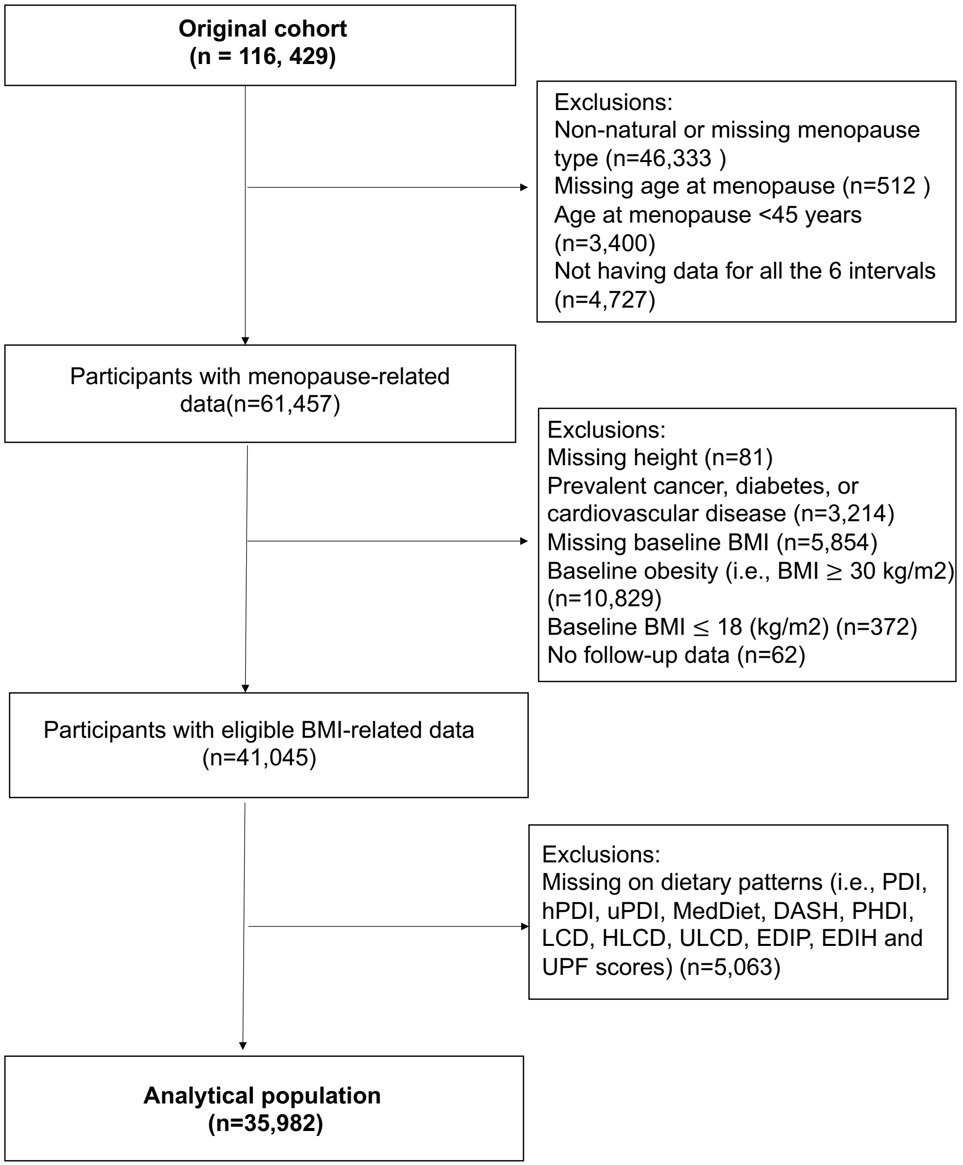

eFigure 3. Sample selection of obesity analysis. BMI, body mass index; DASH, Dietary Approaches to Stop Hypertension; EDIH, empirical dietary index for hyperinsulinemia; EDIP, empirical dietary inflammation pattern; HLCD, healthy low-carbohydrate diet; hPDI, healthy plant-based diet index; LCD, low-carbohydrate diet; MedDiet, Mediterranean diet; MET, metabolic equivalent; PDI, plant-based diet index; PHDI, Planetary Health Diet Index; ULCD, unhealthy low-carbohydrate diet; uPDI, unhealthy plant-based diet index; UPF, ultra-processed food

© 2026 Xia T et al. JAMA Network Open.

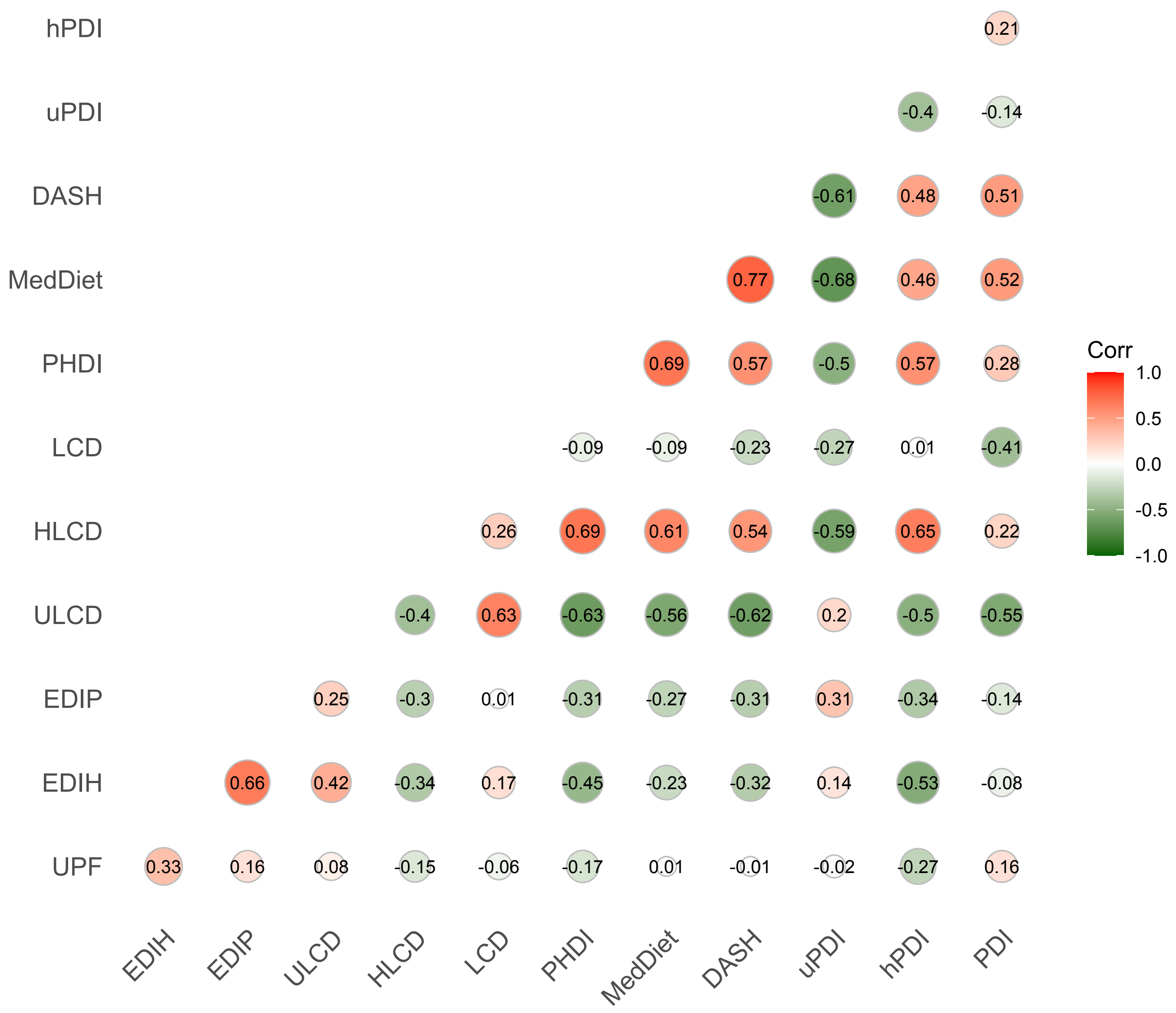

eFigure 4. Pairwise Spearman correlations between dietary pattern scores. DASH, Dietary Approaches to Stop Hypertension; EDIH, empirical dietary index for hyperinsulinemia; EDIP, empirical dietary inflammation pattern; HLCD, healthy low-carbohydrate diet; hPDI, healthy plant-based diet index; LCD, low-carbohydrate diet; MedDiet, Mediterranean diet; MET, metabolic equivalent; PDI, plant-based diet index; PHDI, Planetary Health Diet Index; ULCD, unhealthy low-carbohydrate diet; uPDI, unhealthy plant-based diet index; UPF, ultra-processed food

© 2026 Xia T et al. JAMA Network Open.

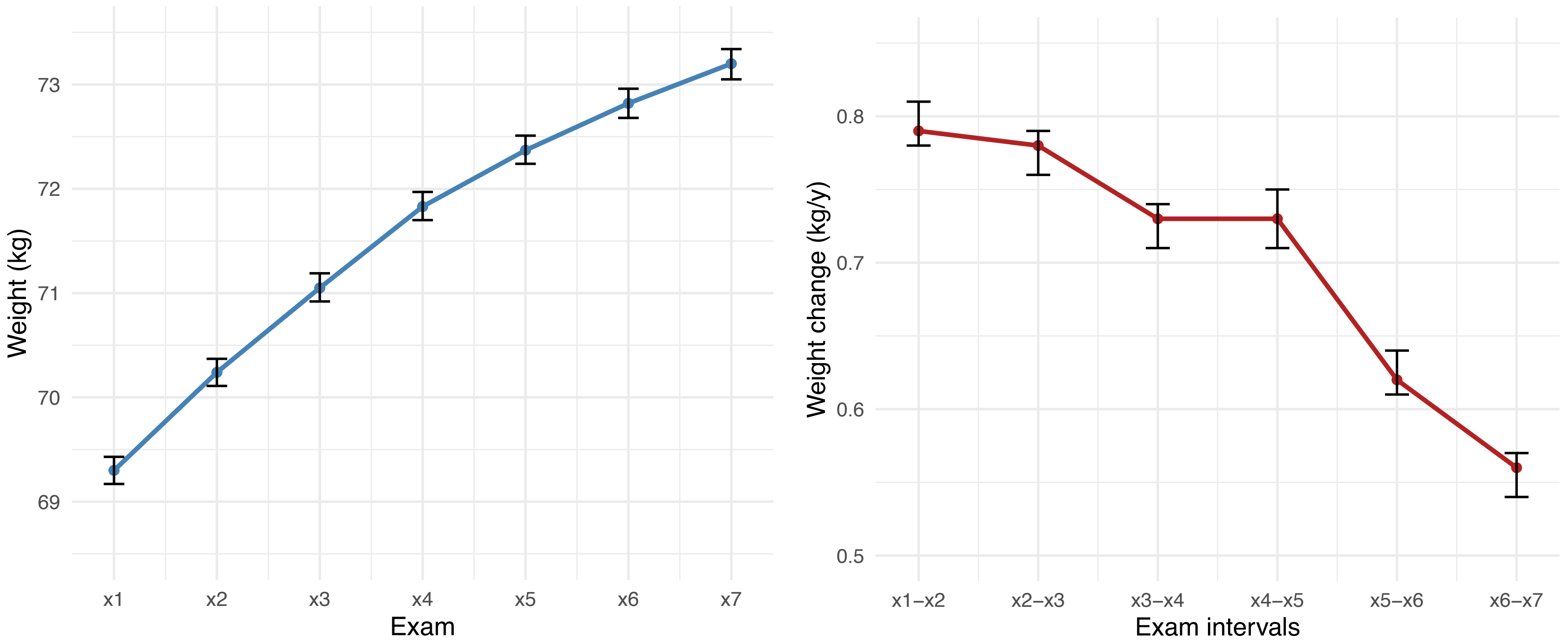

eFigure 5. Weight (kg) and weight gain (kg/year) in each interval across 12 years around menopause. Mean and 95%CI were shown. Menopause was determined when participants reported cessation of menstruation for at least one year (questionnaire x4). Three questionnaires before (x1–x3) and after (x5–x7) menopause captured weight change in six 2-year intervals, with x1 as baseline.

© 2026 Xia T et al. JAMA Network Open.

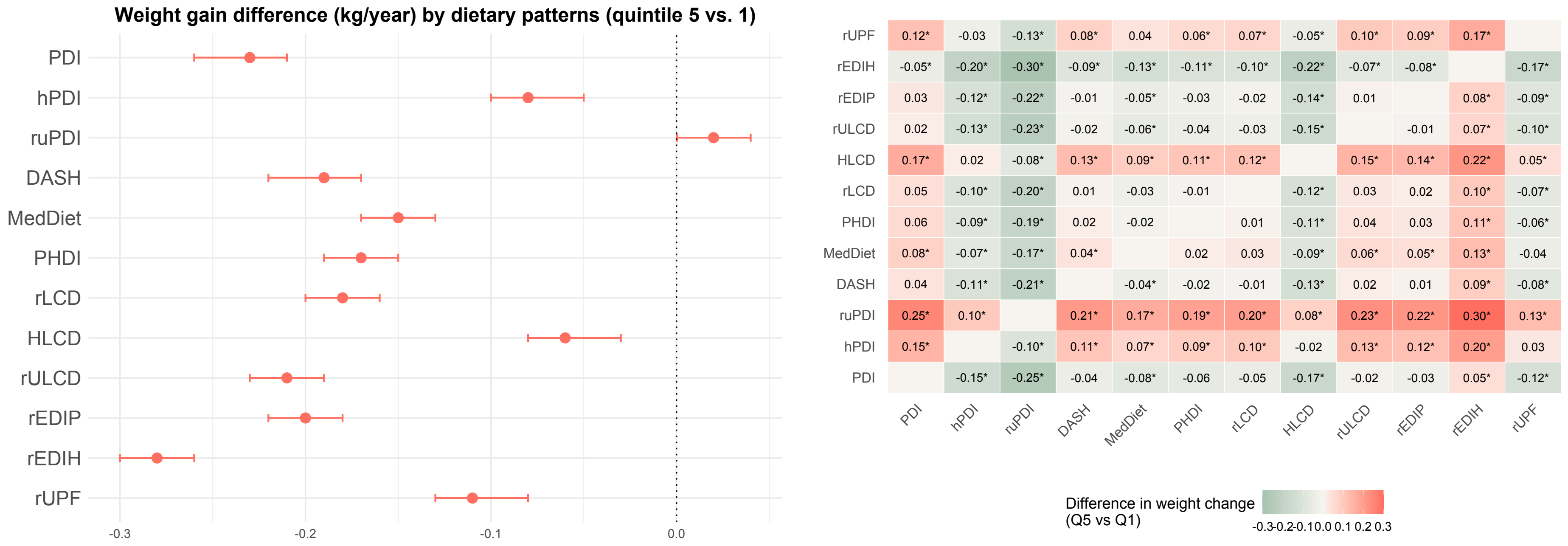

eFigure 6. Associations between dietary patterns and weight gain, and comparison of weight change difference between all pairs of scores. Adjusted mean annualized weight change (kg/year) and 95%CI were reported, which were estimated using generalized estimating equations with repeated measures to account for within-person correlation across follow-up intervals, specifying an unstructured working correlation matrix. Models were adjusted for baseline age (≤45, >45 years), race (White; Asian, American Indian/Alaska Native, Native Hawaiian/Other; Black; Hispanic), marital status (never, ever), income (<50k, 50k–99k, ≥100k, missing), postmenopausal hormone use (never, ever, missing), parity (nulliparous, 1 and 2, or ≥3 births), smoking status (never, ever), alcohol (g/day), total energy intake (<median, ≥median), physical activity (<1000, ≥1000 MET-mins/week), and each interval baseline body mass index (>18 and <25 kg/m2, ≥25 kg/m2). For MedDiet, EDIP, EDIH, the alcohol was not adjusted for. Heatmaps show the weight change difference between all pairs of scores (healthy diet scores and reversed unhealthy diet scores (uPDI, LCD, ULCD, EPIP, EDIH, UPF); quintile 5 vs 1), based on pairwise z-tests. * indicated Bonferroni-adjusted P < 0.05 for comparison. Positive differences are denoted in pink and negative differences in green; a darker color indicates a greater difference. BMI: body mass index; DASH, Dietary Approaches to Stop Hypertension; EDIH, empirical dietary index for hyperinsulinemia; EDIP, empirical dietary inflammation pattern; HLCD, healthy low-carbohydrate diet; hPDI, healthy plant-based diet index; LCD, low-carbohydrate diet; MedDiet, Mediterranean diet; MET, metabolic equivalent; PDI, plant-based diet index; PHDI, Planetary Health Diet Index; rEDIH, reversed empirical dietary index for hyperinsulinemia; rEDIP, reversed empirical dietary inflammation pattern; rLCD, reversed low-carbohydrate diet; rULCD, reversed unhealthy low-carbohydrate diet; ruPDI, reversed unhealthy plant-based diet index; rUPF, reversed and ultra-processed food; ULCD, unhealthy low-carbohydrate diet; uPDI, unhealthy plant-based diet index; UPF, ultra-processed food

© 2026 Xia T et al. JAMA Network Open.

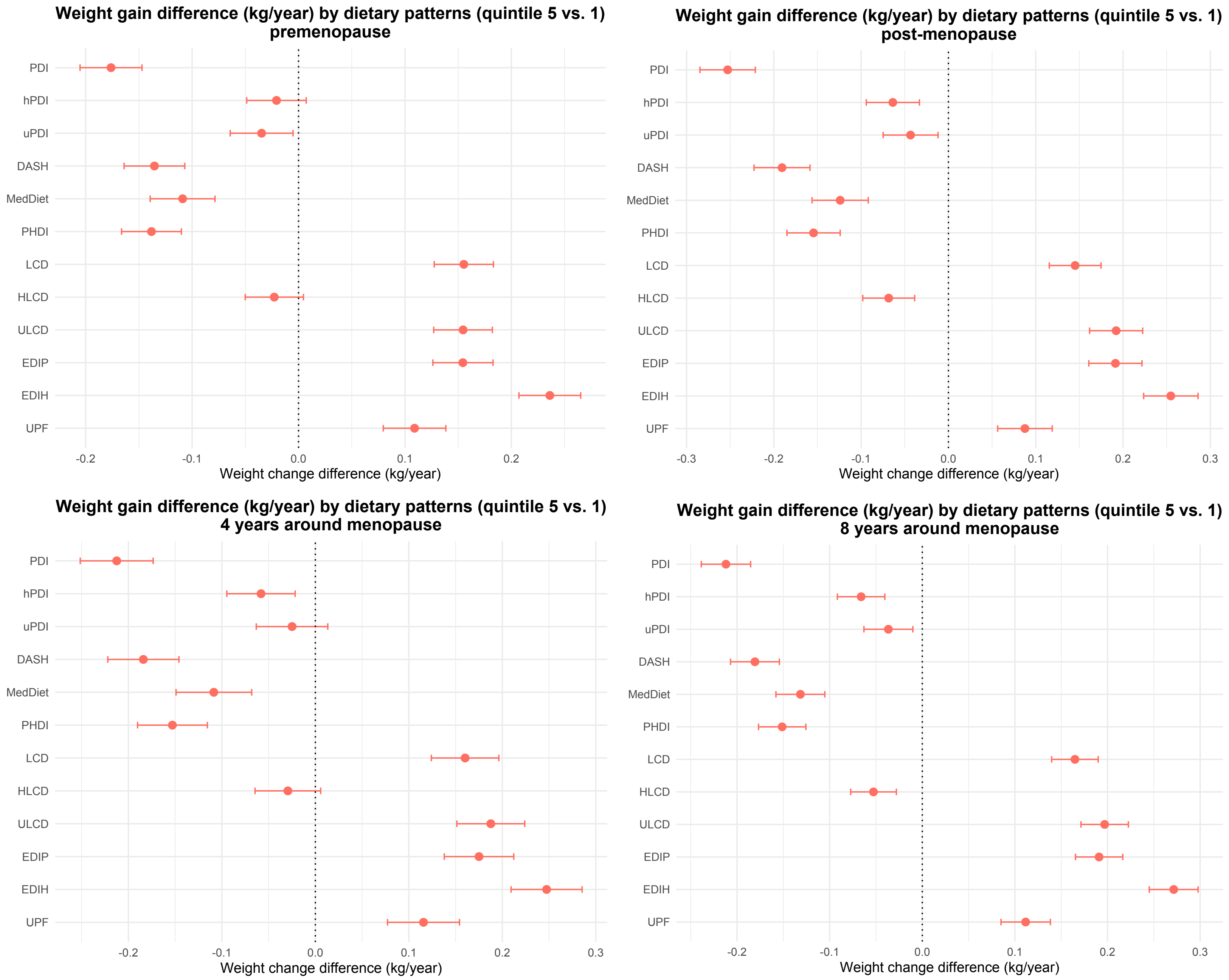

eFigure 7. Associations between dietary patterns and weight gain in women pre- and post-menopause, and within 4-year and 8-year around menopause. Adjusted mean annualized weight change (kg/year) and 95%CI were reported, which were estimated using

© 2026 Xia T et al. JAMA Network Open.

generalized estimating equations with repeated measures to account for within-person correlation across follow-up intervals, specifying an unstructured working correlation matrix. Models were adjusted for baseline age (≤45, >45 years), race (White; Asian, American Indian/Alaska Native, Native Hawaiian/Other; Black; Hispanic), marital status (never, ever), income (<50k, 50k–99k, ≥100k, missing), postmenopausal hormone use (never, ever, missing), parity (nulliparous, 1 and 2, or ≥3 births), smoking status (never, ever), alcohol (g/day), total energy intake (<median, ≥median), physical activity (<1000, ≥1000 MET-mins/week), and each interval baseline body mass index (>18 and <25 kg/m2, ≥25 kg/m2). For MedDiet, EDIP, EDIH, the alcohol was not adjusted for. DASH, Dietary Approaches to Stop Hypertension; EDIH, empirical dietary index for hyperinsulinemia; EDIP, empirical dietary inflammation pattern; HLCD, healthy low-carbohydrate diet; hPDI, healthy plant-based diet index; LCD, low-carbohydrate diet; MedDiet, Mediterranean diet; MET, metabolic equivalent; PDI, plant-based diet index; PHDI, Planetary Health Diet Index; ULCD, unhealthy low-carbohydrate diet; uPDI, unhealthy plant-based diet index; UPF, ultra-processed food

© 2026 Xia T et al. JAMA Network Open.

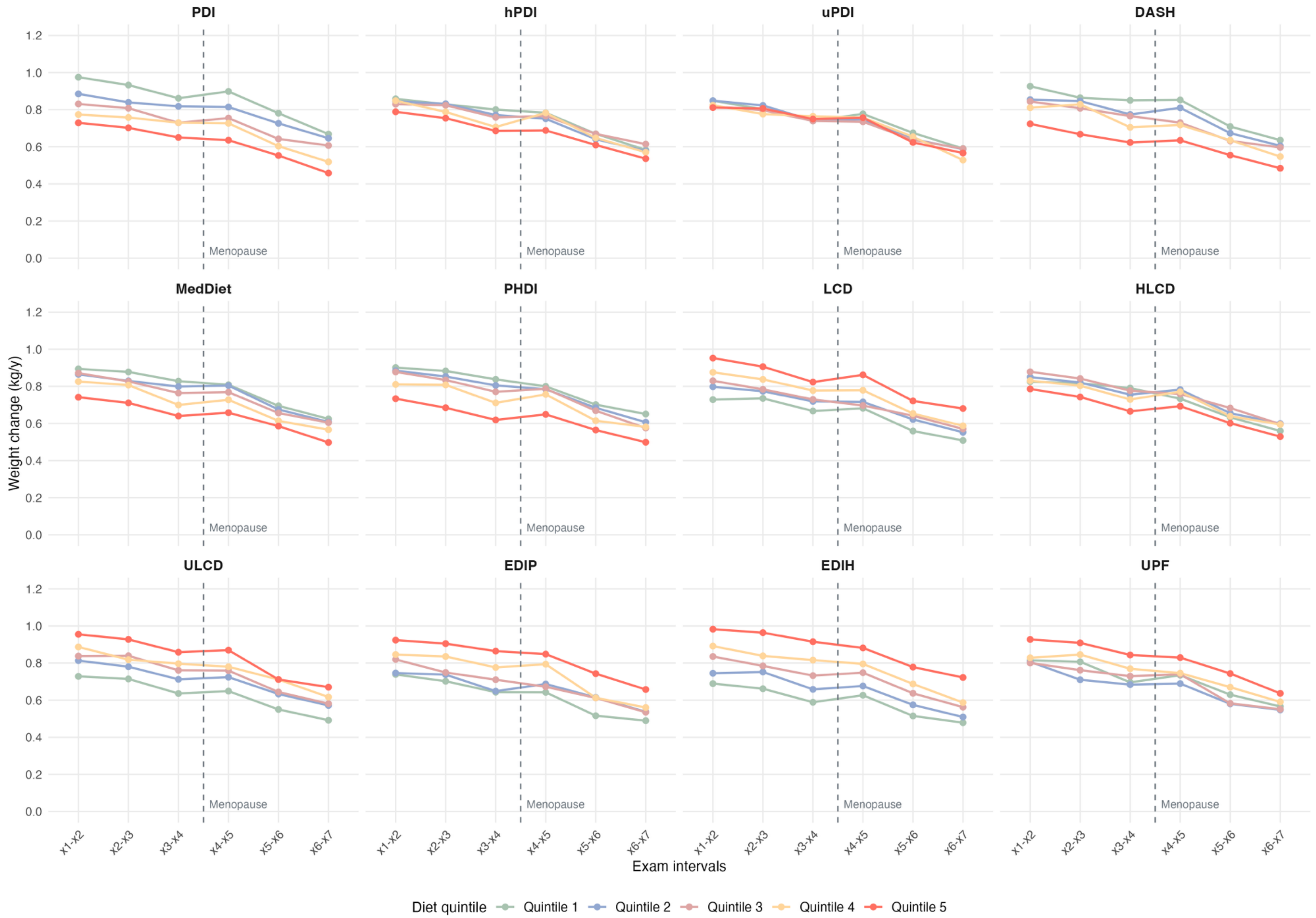

eFigure 8. Annualized weight change by diet patterns in 12 years around menopause. Models were adjusted for baseline age (≤45, >45 years), race (White; Asian, American Indian/Alaska Native, Native Hawaiian/Other; Black; Hispanic), marital status (never,

© 2026 Xia T et al. JAMA Network Open.

ever), income (<50k, 50k–99k, ≥100k, missing), postmenopausal hormone use (never, ever, missing), parity (nulliparous, 1 and 2, or ≥3 births), smoking status (never, ever), alcohol (g/day), total energy intake (<median, ≥median), physical activity (<1000, ≥1000 MET-mins/week), and each interval baseline body mass index (>18 and <25 kg/m2, ≥25 kg/m2). For MedDiet, EDIP, EDIH, the alcohol was not adjusted for. Dietary Approaches to Stop Hypertension; EDIH, empirical dietary index for hyperinsulinemia; EDIP, empirical dietary inflammation pattern; HLCD, healthy low-carbohydrate diet; hPDI, healthy plant-based diet index; LCD, low-carbohydrate diet; MedDiet, Mediterranean diet; MET, metabolic equivalent; PDI, plant-based diet index; PHDI, Planetary Health Diet Index; ULCD, unhealthy low-carbohydrate diet; uPDI, unhealthy plant-based diet index; UPF, ultra-processed food

© 2026 Xia T et al. JAMA Network Open.

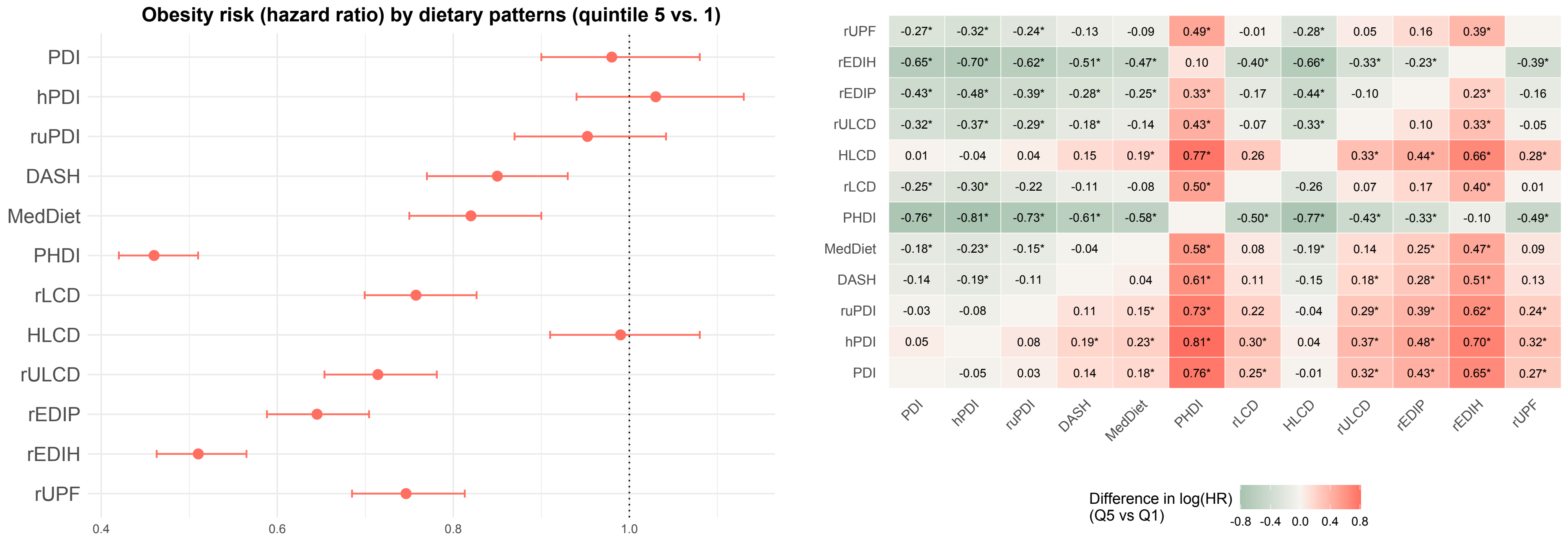

eFigure 9. Associations between dietary patterns and obesity risk, and comparison of difference of log hazard ratio of obesity between all pairs of scores. Cox proportional hazards model was used. Hazard ratio (95% CI), and P-value were reported. Models were adjusted edfor baseline age (≤45, >45 years), race (White; Asian, American Indian/Alaska Native, Native Hawaiian/Other; Black; Hispanic), marital status (never, ever), income (<50k, 50k–99k, ≥100k, missing), postmenopausal hormone use (never, ever, missing), parity (nulliparous, 1 and 2, or ≥3 births), smoking status (never, ever), alcohol (g/day), total energy intake (<median, ≥median), and physical activity (<1000, ≥1000 MET-mins/week), and baseline body mass index (>18 and <25 kg/m^2, ≥25 and <30 kg/m^2). For MedDiet, EDIP, EDIH, the alcohol was not adjusted for. The heatmaps show the difference of log hazard ratio of obesity between all pairs of scores (healthy diet scores and reversed unhealthy diet scores (uPDI, LCD, ULCD, EPIP, EDIH, UPF); quintile 5 vs 1), based on pairwise z-tests. * indicated Bonferroni-adjusted P < 0.05 for comparison. Positive differences are denoted in pink and negative differences in green; a darker color indicates a greater difference. BMI: body mass index; DASH, Dietary Approaches to Stop Hypertension; EDIH, empirical dietary index for hyperinsulinemia; EDIP, empirical dietary inflammation pattern; HLCD, healthy low-carbohydrate diet; hPDI, healthy plant-based diet index; LCD, low-carbohydrate diet; MedDiet, Mediterranean diet; MET, metabolic equivalent; PDI, plant-based diet index; PHDI, Planetary Health Diet Index; rEDIH, reversed empirical dietary index for hyperinsulinemia; rEDIP, reversed empirical dietary inflammation pattern; rLCD, reversed low-carbohydrate diet; rULCD, reversed unhealthy low-carbohydrate diet; ruPDI, reversed unhealthy plant-based diet index; rUPF, reversed and ultra-processed food; ULCD, unhealthy low-carbohydrate diet; uPDI, unhealthy plant-based diet index; UPF, ultra-processed food

© 2026 Xia T et al. JAMA Network Open.

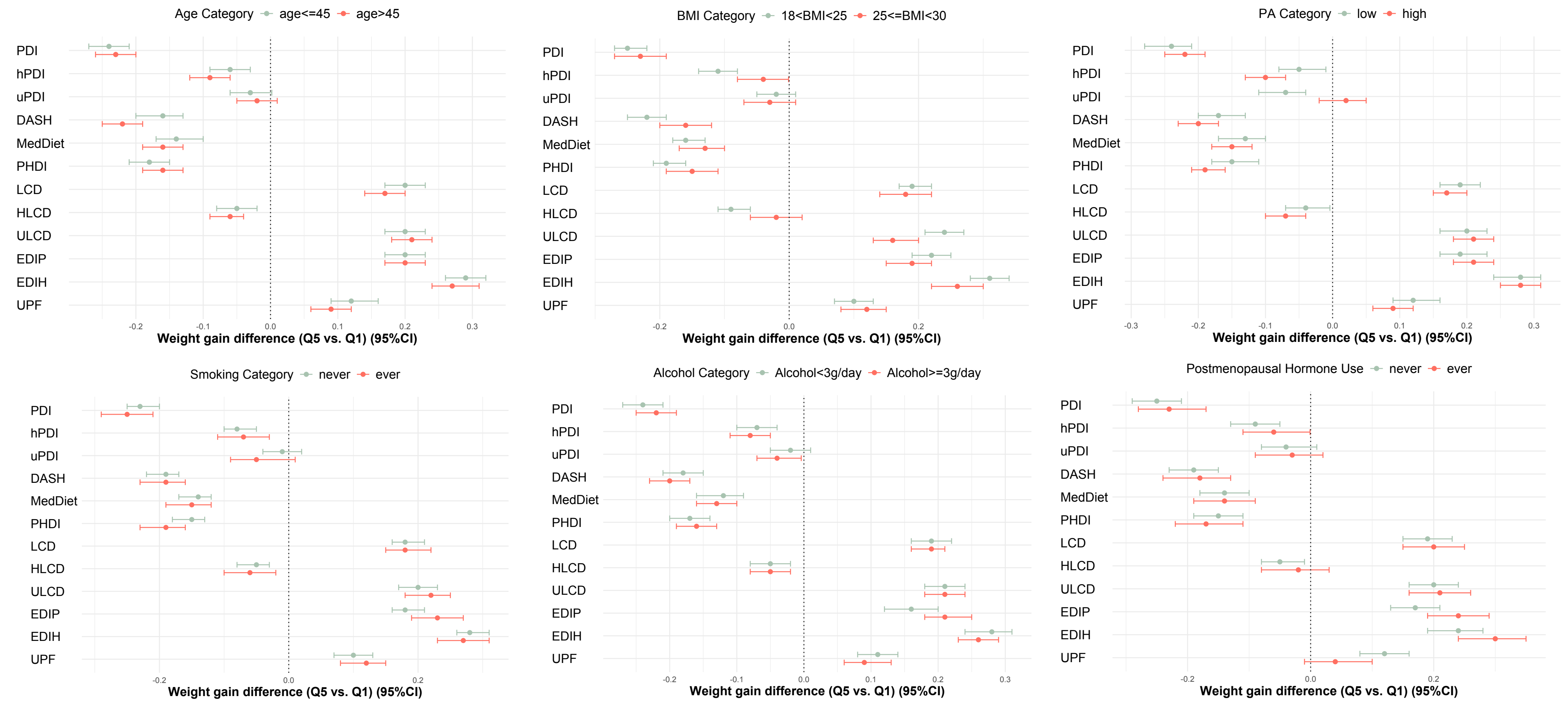

eFigure 10. Associations between dietary patterns (quintile 5 vs. 1) and weight gain in subgroups. Adjusted mean annualized weight change (kg/year) and 95%CI were reported, which were estimated using generalized estimating equations with repeated measures to account for within-person correlation across follow-up intervals, specifying an unstructured working correlation matrix. Models were adjusted for baseline age (≤45, >45 years),), race (White; Asian, American Indian/Alaska Native, Native Hawaiian/Other; Black; Hispanic), marital status (never, ever), income (<50k, 50k–99k, ≥100k, missing), postmenopausal hormone use (never, ever, missing), parity (nulliparous, 1 and 2, or ≥3 births), smoking status (never, ever), alcohol (g/day), total energy intake (<median, ≥median), physical activity (<1000, ≥1000 MET-mins/week), and each interval baseline body mass index (>18 and <25 kg/m2, ≥25 kg/m2). For MedDiet, EDIP, EDIH, the alcohol was not adjusted for. When the association was stratified by one variable, that variable was not adjusted for in the model. Effect modification was tested using Wald χ^2. PA category: low (PA<1000 MET-mins/week), high (PA≥1000 MET-mins/week). BMI, body mass index; CI, confidence interval; DASH, Dietary Approaches to Stop Hypertension; EDIH, empirical dietary index for hyperinsulinemia; EDIP, empirical dietary inflammation pattern; HLCD, healthy low-carbohydrate diet; hPDI, healthy plant-based diet index; LCD, low-carbohydrate diet; MedDiet, Mediterranean diet; MET, metabolic equivalent; PA, physical activity; PDI, plant-based diet index; PHDI, Planetary Health Diet Index; ULCD, unhealthy low-carbohydrate diet; uPDI, unhealthy plant-based diet index; UPF, ultra-processed food

© 2026 Xia T et al. JAMA Network Open.

eReferences

1. El Khoudary SR, Aggarwal B, Beckie TM, et al. Menopause Transition and Cardiovascular Disease Risk: Implications for Timing of Early Prevention: A Scientific Statement From the American Heart Association. *Circulation*. 2020;142(25). doi:10.1161/CIR.0000000000000912
2. Tepper PG, Randolph JF, McConnell DS, et al. Trajectory Clustering of Estradiol and Follicle-Stimulating Hormone during the Menopausal Transition among Women in the Study of Women's Health across the Nation (SWAN). *The Journal of Clinical Endocrinology & Metabolism*. 2012;97(8):2872-2880. doi:10.1210/jc.2012-1422
3. Yuan M, Hu FB, Li Y, et al. Dairy Foods, Weight Change, and Risk of Obesity During the Menopausal Transition. *The Journal of Nutrition*. 2023;153(3):811-819. doi:10.1016/j.tjnut.2023.01.001
4. Jeor STSt, Brunner RL, Harrington ME, et al. Who Are the Weight Maintainors? *Obesity Research*. 1995;3(S2). doi:10.1002/j.1550-8528.1995.tb00471.x
5. ST JEOR ST, BRUNNER RL, HARRINGTON ME, et al. A classification system to evaluate weight maintainers, gainers, and losers. Journal of the American Dietetic Association. 1997;97(5):481-488. doi:10.1016/s0002-8223(97)00126-0
6. Satija A, Bhupathiraju SN, Rimm EB, et al. Plant-Based Dietary Patterns and Incidence of Type 2 Diabetes in US Men and Women: Results from Three Prospective Cohort Studies. Moore SC, ed. *PLoS Med*. 2016;13(6):e1002039. doi:10.1371/journal.pmed.1002039
7. Fung TT, Pan A, Hou T, et al. Long-Term Change in Diet Quality Is Associated with Body Weight Change in Men and Women. *The Journal of Nutrition*. 2015;145(8):1850-1856. doi:10.3945/jn.114.208785
8. Sawicki CM, Ramesh G, Bui L, et al. Planetary health diet and cardiovascular disease: results from three large prospective cohort studies in the USA. *The Lancet Planetary Health*. 2024;8(9):e666-e674. doi:10.1016/S2542-5196(24)00170-0
9. Liu B, Hu Y, Rai SK, Wang M, Hu FB, Sun Q. Low-Carbohydrate Diet Macronutrient Quality and Weight Change. *JAMA Netw Open*. 2023;6(12):e2349552. doi:10.1001/jamanetworkopen.2023.49552
10. Tabung FK, Wang W, Fung TT, et al. Development and validation of empirical indices to assess the insulinaemic potential of diet and lifestyle. *Br J Nutr*. 2016;116(10):1787-1798. doi:10.1017/S0007114516003755
11. Tabung FK, Smith-Warner SA, Chavarro JE, et al. Development and Validation of an Empirical Dietary Inflammatory Index. *The Journal of Nutrition*. 2016;146(8):1560-1570. doi:10.3945/jn.115.228718
12. Chen Z, Khandpur N, Desjardins C, et al. Ultra-Processed Food Consumption and Risk of Type 2 Diabetes: Three Large Prospective U.S. Cohort Studies. *Diabetes Care*. 2023;46(7):1335-1344. doi:10.2337/dc22-1993

© 2026 Xia T et al. JAMA Network Open.
