# Supplementary material for: Optimal Dietary Patterns for Lower Weight Gain and Risk of Obesity Surrounding Menopause
Source: JAMA Netw Open. 2026 May 20;9(5):e2613102. doi: 10.1001/jamanetworkopen.2026.13102 (PMC13191392; doi:10.1001/jamanetworkopen.2026.13102)
Supplement: Supplement 2. — Data Sharing Statement [file jamanetwopen-e2613102-s002.pdf]

Data Sharing Statement

Xia. Optimal Dietary Patterns for Lower Weight Gain and Risk of Obesity Surrounding Menopause. *JAMA Netw Open*. Published May 18, 2026.
doi:10.1001/jamanetworkopen.2026.13102

Data
Data available: No
